# Proto-oncogene Src links lipogenesis via lipin-1 to breast cancer malignancy

**DOI:** 10.1038/s41467-020-19694-w

**Published:** 2020-11-17

**Authors:** Lintao Song, Zhihua Liu, Hui-Hui Hu, Ying Yang, Terytty Yang Li, Zhi-Zhong Lin, Jing Ye, Jianing Chen, Xi Huang, Dong-Tai Liu, Jing Zhou, Yiran Shi, Hao Zhao, Changchuan Xie, Lanfen Chen, Erwei Song, Shu-Yong Lin, Sheng-Cai Lin

**Affiliations:** 1 State Key Laboratory of Cellular Stress Biology, School of Life Sciences, Xiamen University, 361102 Fujian, China; 2 Center of Intestinal Barrier and Fecal Microbiota Transplantation, The Fifth Affiliated Hospital of Guangzhou Medical University, 510700 Guangdong, China; 3 Department of Pathology, Xijing Hospital, Fourth Military Medical University, 710000 Shaanxi, China; 4 Breast Tumor Center, Sun Yat-Sen Memorial Hospital, Sun Yat-Sen University, 510120 Guangzhou, China

**Keywords:** Breast cancer, Cancer metabolism, Phosphorylation

## Abstract

Increased lipogenesis has been linked to an increased cancer risk and poor prognosis; however, the underlying mechanisms remain obscure. Here we show that phosphatidic acid phosphatase (PAP) lipin-1, which generates diglyceride precursors necessary for the synthesis of glycerolipids, interacts with and is a direct substrate of the Src proto-oncogenic tyrosine kinase. Obesity-associated microenvironmental factors and other Src-activating growth factors, including the epidermal growth factor, activate Src and promote Src-mediated lipin-1 phosphorylation on Tyr398, Tyr413 and Tyr795 residues. The tyrosine phosphorylation of lipin-1 markedly increases its PAP activity, accelerating the synthesis of glycerophospholipids and triglyceride. Alteration of the three tyrosine residues to phenylalanine (3YF-lipin-1) disables lipin-1 from mediating Src-enhanced glycerolipid synthesis, cell proliferation and xenograft growth. Re-expression of 3YF-lipin-1 in PyVT;*Lpin1*^*−/−*^ mice fails to promote progression and metastasis of mammary tumours. Human breast tumours exhibit increased p-Tyr-lipin-1 levels compared to the adjacent tissues. Importantly, statistical analyses show that levels of p-Tyr-lipin-1 correlate with tumour sizes, lymph node metastasis, time to recurrence and survival of the patients. These results illustrate a direct lipogenesis-promoting role of the pro-oncogenic Src, providing a mechanistic link between obesity-associated mitogenic signaling and breast cancer malignancy.

## Abstract (web summary)

Altered lipid metabolism has been associated with tumour malignancy, but underlying mechanisms are not clear. Here the authors show that proto-oncogene Src interacts and phosphorylates metabolic enzyme phosphatidic acid phosphatase LPIN1 (lipin-1) to promote growth and metastasis in breast cancer.

## Introduction

The cellular proto-oncogene Src (c-Src) is a non-receptor tyrosine kinase, regulating various cellular processes, including those involved in cell proliferation, differentiation, survival and migration^1,2^. Aberrantly activated Src has been known to be a potent oncogenic protein^3^. It is normally maintained in a catalytically inactive conformation by the intramolecular interaction of its carboxyl-terminal phosphotyrosine (Tyr530) with the N-terminal Src homology 2 (SH2) domain^1^. Engagement of receptor tyrosine kinases (RTKs) with pro-mitogenic growth factors such as EGF and PDGF leads to the dephosphorylation of Y530 and consequential de-inhibition of Src^4,5^. Activated Src then autophosphorylates tyrosine 416 residue (Tyr416, Tyr419 of human Src) in the kinase domain, enabling it to target a variety of substrates^1^. Mutations at the Y416 residue or within encompassing segments have been known to cause constitutive activation.

Cancer cells exhibit not only abnormally high demand for glucose and glutamine^6^ but also altered lipid metabolism such as elevated lipogenesis, increased fatty acid uptake. Lipid metabolism has an especially high impact on breast cancer cells^7,8^, as these cancer cells are often surrounded by a large number of adipocytes that actively undergo triglyceride (TAG) cycle and generate a fatty acid-rich environment. Fatty acids are consumed for energy production through β-oxidation, provide building blocks for phospholipids and act as extrinsic stimuli for cellular growth as well^9–11^.

Phosphatidic acid phosphatase *LPIN1* (lipin-1) possesses a dual function as a metabolic enzyme and a transcriptional cofactor for master regulators of lipid metabolism, including peroxisome proliferator-activated receptor α (PPARα)^12,13^ and sterol regulatory element-binding proteins (SREBPs)^14^, which in turn regulate other metabolic pathways such as fatty acid oxidation and de novo lipogenesis. As a metabolic enzyme for glycerolipid synthesis, lipin-1 catalyses the reaction of removing the phosphate group from phosphatidic acids (PA) to yield diacylglycerols (DAG) that in turn can be partitioned into the synthesis of TAGs or glycerophospholipids depending on the downstream enzymes^15,16^. Regulatory mechanisms have been identified for the posttranslational modifications of lipin-1, including phosphorylation^17^ and acetylation^18^. Lipin-1 has been found to be aberrantly upregulated in certain types of cancer cells, and its PAP activity is required for the survival of these cells^19–22^. However, the epistatic interactions between Src and lipin-1, as well as the functional linkage between oncogenic signalling and glycerolipid synthesis in vivo, remain obscure. In this study, through screening for lipin-1-interacting proteins, we found that the Src proto-oncogene protein interacts with and phosphorylates lipin-1. We have demonstrated that the PAP activity of lipin-1 is greatly increased after tyrosine phosphorylation by Src. We have provided evidence that pro-mitogenic growth factors signal to lipin-1 in an Src-dependent manner. Moreover, unphosphorylable lipin-1 is unable to promote growth and metastasis of breast cancer spontaneously developed in PyVT;*Lpin1*^*−/−*^ mice in vivo. Our findings thus reveal that upregulating glycerolipid synthesis is an integral part of the tumour-promoting ability of Src, directly linking lipogenesis to tumour malignancy.

## Results

### Src phosphorylates lipin-1 upon mitogenic stimulation

**Fig. 1:**
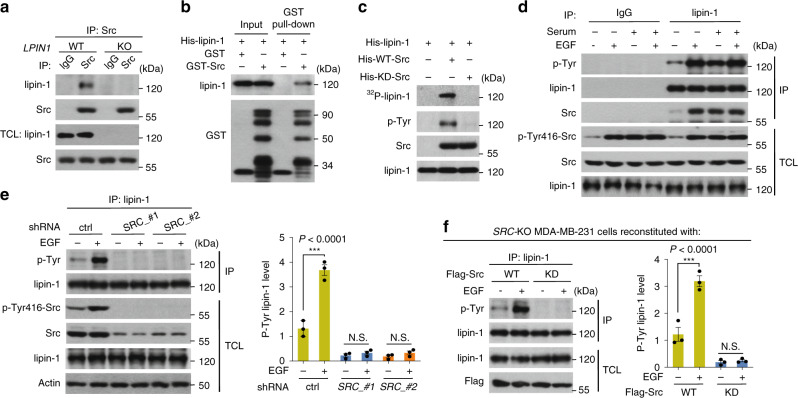
Src directly interacts with and phosphorylates lipin-1 upon mitogenic stimulation. **a** Interaction between endogenous lipin-1 and Src. Src in wild-type (WT) and *LPIN1-*knockout (KO) MDA-MB-231 cells were immunoprecipitated (IP), followed by immunoblotting with antibodies indicated. TCL, total cell lysate. **b** GST pull-down assay was performed by using bacterially purified His-lipin-1 and GST or GST-Src, followed by immunoblotting. **c** Lipin-1 was phosphorylated by Src in vitro. Bacterially expressed His-lipin-1 was incubated with His-WT-Src or His-KD-Src (a kinase-dead form of Src) in a kinase assay buffer, followed by immunoblotting. **d** Lipin-1 tyrosine phosphorylation stimulated by serum, or/and EGF. MDA-MB-231 cells were maintained in a serum-free medium for 4 h, and then treated with or without EGF, serum or serum plus EGF for additional 30 min. Cell lysates were analysed by immunoblotting with antibodies indicated. **e** Increased Src-dependent tyrosine phosphorylation of lipin-1 after EGF treatment. MDA-MB-231 cells expressing shRNA against *SRC* or *Renilla* as a control were maintained in a serum-free medium for 4 h, followed by stimulation with or without EGF for 30 min. Lipin-1 was immunoprecipitated, followed by immunoblotting. Quantification of the ratio of phosphorylated lipin-1 to total lipin-1 is displayed as a scatter plot. P-Tyr-lipin-1, phosphorylation of lipin-1 on tyrosine. **f** The tyrosine phosphorylation of lipin-1 in *SRC*-KO MDA-MB-231 cells reconstituted with Src. After 30 min of EGF incubation, *SRC*-KO MDA-MB-231 cells stably expressing Flag-tagged WT-Src or KD-Src were lysed and subjected to IP with anti-lipin-1 antibody. Quantification of the ratio of phosphorylated lipin-1 to total lipin-1 is displayed as a scatter plot. **e**, **f** were quantified in each biological replicate (*n* = 3); data are mean ± s.e.m.; ordinary two-way ANOVA, followed by Sidak in **e**, **f**; ***P < 0.001, N.S. not significant. Source data are provided as a Source Data file.

To identify potential lipin-1 interacting proteins, we first replaced the endogenous lipin-1 with Flag-tagged counterpart in the MDA-MB-231 cell line of breast cancer origin by using the CRISPR/Cas9 technique (Supplementary Fig. 1a, b). The Flag-tagged lipin-1 was immunoprecipitated, followed by mass spectrometry analysis. Among the co-immunoprecipitated proteins, Src protein was identified as a potential new lipin-1-associated protein (Supplementary Fig. 2a, b). The interaction was verified, showing that the endogenous Src was co-precipitated with lipin-1 in wild-type MDA-MB-231 cells, but not in *LPIN1-*knockout cells (Fig. 1a). We also performed co-immunoprecipitation in HEK293T cells transfected with Src and lipin-1 (Supplementary Fig. 2c, d). In vitro GST pull-down assay indicated a direct interaction between lipin-1 and Src (Fig. 1b and Supplementary Fig. 2e). Intriguingly, the interaction was diminished by Src inhibitors, SKI-606 and Dasatinib (Supplementary Fig. 2f, g); KD-Src (kinase-dead) and DN-Src (dominant-negative) mutants showed much-compromised interaction with lipin-1 (Supplementary Fig. 2h). Conversely, markedly higher levels of lipin-1 were co-precipitated with a constitutively active form of Src (Y529F-Src) (Supplementary Fig. 2h). These data indicated that an active structure or conformation of the Src kinase appears to be essential for its interaction with lipin-1.

We then asked whether lipin-1 is a direct phosphorylation substrate of Src. Enzymes involved in the TAG synthesis pathway were co-transfected with Src into HEK293T cells and were then immunoprecipitated (Supplementary Fig. 3a). As shown in Supplementary Fig. 3b, only lipin-1, but not the other enzymes, was tyrosine phosphorylated. After treatment with calf-intestinal alkaline phosphatase (CIP), the phosphorylated tyrosine (p-Tyr) signal disappeared (Supplementary Fig. 3c). In vitro kinase assays confirmed that Src could directly phosphorylate lipin-1 (Fig. 1c). We thus examined whether growth factors would increase Src-mediated lipin-1 tyrosine phosphorylation by immunoblotting with a general antibody against phosphotyrosine after immunoprecipitation of lipin-1. In serum-deprived MDA-MB-231 cells treated with or without serum and growth factor (EGF), lipin-1 tyrosine phosphorylation was markedly increased, concurring with elevated phosphorylation of Src on Tyr416, which is known to be an activating modification for Src^1^ (Fig. 1d). Similar results were obtained when breast cancer cells were treated with EGF, PDGF and IGF-1 (Supplementary Fig. 3d–h). EGF treatment failed to increase the levels of p-Tyr-lipin-1 in *SRC*-knockdown MDA-MB-231 or MDA-MB-468 cells (Fig. 1e and Supplementary Fig. 3i). In addition, treatment of cells with the Src inhibitor Dasatinib abrogated EGF-increased lipin-1 phosphorylation (Supplementary Fig. 3j). Furthermore, reconstitution of *SRC*-KO cells with WT-Src, but not KD-Src, restored the EGF-increased tyrosine phosphorylation of lipin-1 (Fig. 1f and Supplementary Fig. 3k, l). These results illustrated that signalling from growth factors to Src can stimulate tyrosine phosphorylation on lipin-1.

### Tyrosine phosphorylation of lipin-1 promotes glycerolipid synthesis

**Fig. 2:**
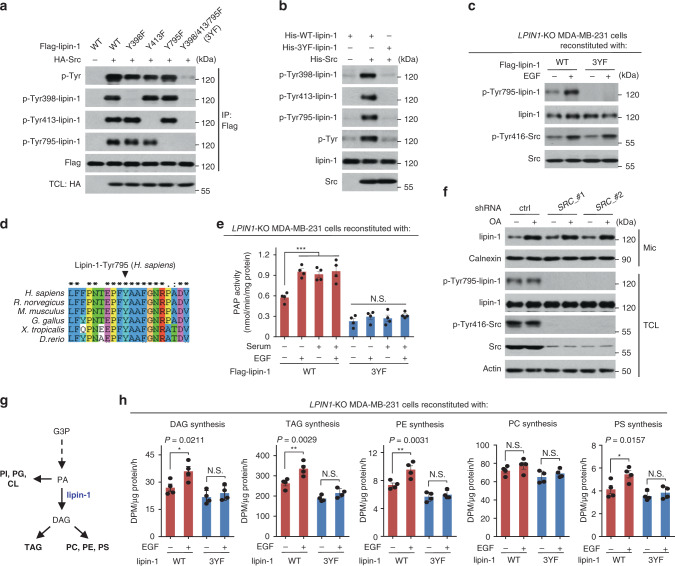
Src-mediated tyrosine phosphorylation of lipin-1 is essential for PAP activity of lipin-1 and glycerolipid synthesis. **a** Identification of phosphorylation sites on lipin-1. Flag-WT-lipin-1 or its tyrosine-to-phenylalanine (YF) mutants was co-expressed with or without Src in HEK293T cells. Cells were lysed and subjected to IP against Flag, followed by immunoblotting. **b** 3YF-lipin-1 mutant fails to be phosphorylated by Src in vitro. Bacterially expressed His-tagged WT-lipin-1 or its 3YF mutant was incubated with His-Src in a kinase assay buffer, followed by immunoblotting. **c** 3YF-lipin-1 mutant fails to be phosphorylated in EGF-stimulated in MDA-MB-231 cells. *LPIN1*-KO MDA-MB-231 cells stably expressing Flag-tagged WT-lipin-1 or 3YF-lipin-1 were maintained in a serum-free medium for 4 h, followed with or without EGF treatment. **d** Sequence alignment of the residues flanking Tyr795 across different species. Arrowhead points to the tyrosine residues corresponding to the Tyr795 residue in human lipin-1. **e** PAP activity in *LPIN1*-KO MDA-MB-231 cells reconstituted with WT-lipin-1 or 3YF-lipin-1. Cells were maintained in a serum-free medium for 4 h, and then treated with or without serum or EGF, followed by immunoprecipitation with anti-Flag. The enzymatic activities of immunoprecipitated Flag-lipin-1 were examined. **f** Knockdown of *SRC* does not affect oleic acid (OA)-induced lipin-1 translocation to the endoplasmic reticulum (ER). MDA-MB-231 cells expressing shRNA against *SRC* or *Renilla* as a control were maintained in complete medium containing 10% FBS and treated with or without OA for 2 h and homogenised and subjected to ultracentrifugation to collect microsome fractions, followed by immunoblotting. Calnexin, microsomal (Mic) marker. **g** Schematic diagram of phospholipids synthesised from glycerol-3-phosphate in mammalian cells. **h** DAG, TAG and phospholipid synthesis rates of *LPIN1*-KO MDA-MB-231 cells reconstituted with WT-lipin-1 or 3YF-lipin-1. Lipids from cells treated with ^3^H-labelled OA and EGF were extracted and resolved by thin-layer chromatography (TLC), followed by quantification with scintillation counting. DAG diglyceride, TAG triglyceride, PE phosphatidylethanolamine, PC phosphatidylcholine, PS phosphatidylserine. **e**, **h** were quantified in each independent experiment (*n* = 4). Data are mean ± s.e.m.; ordinary two-way ANOVA, followed by Tukey in **e**, or by Sidak in **h**; ***P < 0.001, N.S. not significant. Source data are provided as a Source Data file.

To identify the phosphorylated tyrosine residues, lipin-1 phosphorylated in vitro by Src was subjected to mass spectrometry. Multiple phosphorylated candidate tyrosine residues of lipin-1 were identified (Supplementary Fig. 4a, b). We then created mutants carrying alteration of those tyrosine residues to phenylalanine singly or in combination. It was found that the combined mutation of Tyr398, Tyr413 and Tyr795 (3YF) on lipin-1 strongly abolished Src-catalysed p-Tyr signal detected in lipin-1 immunoprecipitates (Fig. 2a–c). Sequence alignment indicates that these tyrosine residues in lipin-1 are highly conserved across different species (Fig. 2d and Supplementary Fig. 4c). We then raised polyclonal antibodies that specifically recognise lipin-1 phosphorylated at each of the three different tyrosine residues. By using these antibodies, we verified that the three tyrosine residues of lipin-1 are indeed phosphorylated in an Src-dependent manner in culture cells and in vitro (Fig. 2a, b). Moreover, compared to WT-lipin-1, the unphosphorylable 3YF-lipin-1 reintroduced to *LPIN1*-KO or -KD (knockdown) cells showed little, if any, p-Tyr signal even after growth factor treatment (Fig. 2c and Supplementary Fig. 4d, e). These data clearly demonstrated that lipin-1 is a bona fide substrate of Src. Considering the potential functional similarity of Src-family kinases, we also examined the effect on lipin-1 of three other broadly expressed Src-family members, Yes1, Fyn and Lyn. We found that Fyn and Lyn could also phosphorylate lipin-1 albeit at much lower efficiency, while Yes1 is unable to phosphorylate lipin-1 (Supplementary Fig. 4f).

It has been known that mTORC1 inhibits the PAP activity of lipin-1 via serine/threonine phosphorylation^14,23,24^. We thus tested whether mTORC1-mediated phosphorylation would affect Src-mediated tyrosine phosphorylation of lipin-1 or vice versa. Under insulin- or EGF-stimulated conditions, Src-mediated tyrosine phosphorylation of lipin-1 was not affected by the mTOR inhibitor rapamycin or Torin 1, although mTOR inhibitors effectively blocked lipin-1 serine/threonine phosphorylation (Supplementary Fig. 5a–f). Similarly, the Src inhibitors did not affect mTORC1-mediated serine phosphorylation (Supplementary Fig. 5c–f), indicating that Src and mTORC1 regulate lipin-1 via independent mechanisms.

To find out the effect of Src-mediated tyrosine phosphorylation on lipin-1, we investigated the PAP activity of lipin-1 before and after phosphorylation by Src. WT-lipin-1 showed higher enzymatic activity than 3YF-lipin-1 in cells cultured in normal complete medium (Supplementary Fig. 6a). EGF treatment or Src overexpression increased the PAP activity of WT-lipin-1 (Fig. 2e and Supplementary Fig. 6a). By contrast, 3YF-lipin-1 failed to respond to such stimulation (Fig. 2e and Supplementary Fig. 6a). Of note, 3YE-lipin-1 and 3YD-lipin-1, putative p-Tyr mimetic mutants after changing Y to E or D, showed similar catalytic activity as 3YF-lipin-1, indicating that these substitutions cannot mimic Src-mediated tyrosine phosphorylation in lipin-1 (Supplementary Fig. 6a). We then determined the Michaelis constant (*K*_m_) values of lipin-1, and found that Src-mediated phosphorylation benefits lipin-1 PAP activity likely by increasing the binding affinity of lipin-1 with its substrate PA (Supplementary Fig. 6b). It was reported that, in response to fatty acid, lipin-1 becomes serine/threonine dephosphorylated and translocates from cytoplasm to endoplasmic reticulum (ER)-associated membranes where the glycerolipid biosynthesis takes place^17^. We thus examined whether fatty acid-induced ER localisation of lipin-1 is affected after tyrosine phosphorylation by Src. It was found that fatty acid-induced ER translocation of lipin-1 was not regulated by Src, indicating that the lipin-1 translocation is not influenced by tyrosine phosphorylation (Fig. 2f and Supplementary Fig. 6c, d), but likely through its acetylation as shown previously^18^. Collectively, these results suggest that Src-mediated tyrosine phosphorylation of lipin-1 mainly upregulates the PAP activity. Consistent with the proteomic data showing that lipin-1 is the major PAP in human breast cancer samples^25^, knockdown of *LPIN1*, but not its homologues *LPIN2* or *LPIN3*, virtually abolished the PAP activity in breast cancer cell lines (Supplementary Fig. 6e, f), confirming that lipin-1 accounts for the majority of PAP activity in these cells.

We then investigated whether lipin-1 tyrosine phosphorylation by Src directly affects glycerolipid synthesis in MDA-MB-231 and MDA-MB-468 cells by using ^3^H-labelled oleic acid (OA) as a tracer. When cultured in serum-free medium, EGF significantly accelerated the synthesis rates of DAG, TAG, phosphatidylethanolamine (PE) and phosphatidylserine (PS) in these two breast cancer cell lines when normalised to protein abundance (Fig. 2g, h and Supplementary Fig. 7a, b). The mean values in the synthesis rate of phosphatidylcholine (PC) were reproducibly increased as well, while the level of phosphatidylinositol (PI) was decreased (likely due to PA being driven to DAG as diagramed in Fig. 2g), albeit with a lack of statistical significance (Fig. 2h and Supplementary Fig. 7a, b). The EGF-induced glycerolipid synthesis was markedly suppressed by the Src inhibitor Dasatinib (Supplementary Fig. 7c, d). Moreover, compared to WT-lipin-1, the unphosphorylable 3YF-lipin-1 exhibited much lower ability to mediate the induction by EGF of the synthesis of TAG and phospholipids (PE and PS) when reintroduced to *LPIN1*-KO MDA-MB-231 cells or *LPIN1*-KD MDA-MB-468 cells (Fig. 2h and Supplementary Fig. 7b). The dependence of glycerolipid synthesis on Src and tyrosine phosphorylation of lipin-1 was also demonstrated in cells cultured in normal complete medium (Supplementary Fig. 7e, f). Specifically, depletion of Src in WT-lipin-1 cells significantly impeded the synthesis of TAG, PE, PC and PS, while moderately increased PI synthesis (Supplementary Fig. 7g, h), and WT-lipin-1 but not the unphosphorylable 3YF-lipin-1 increased the synthesis rates of these lipids (Fig. 2h and Supplementary Fig. 7b, e, f). In addition to the PAP activity, lipin-1 also possesses transcriptional coactivator activity and has been implicated in the regulation of fatty acid oxidation. By contrast, the regulatory role of lipin-1 in fatty acid oxidation was not affected by Src (Supplementary Fig. 7i).

### Proliferation of breast cancer cells depends on tyrosine phosphorylation of lipin-1

**Fig. 3:**
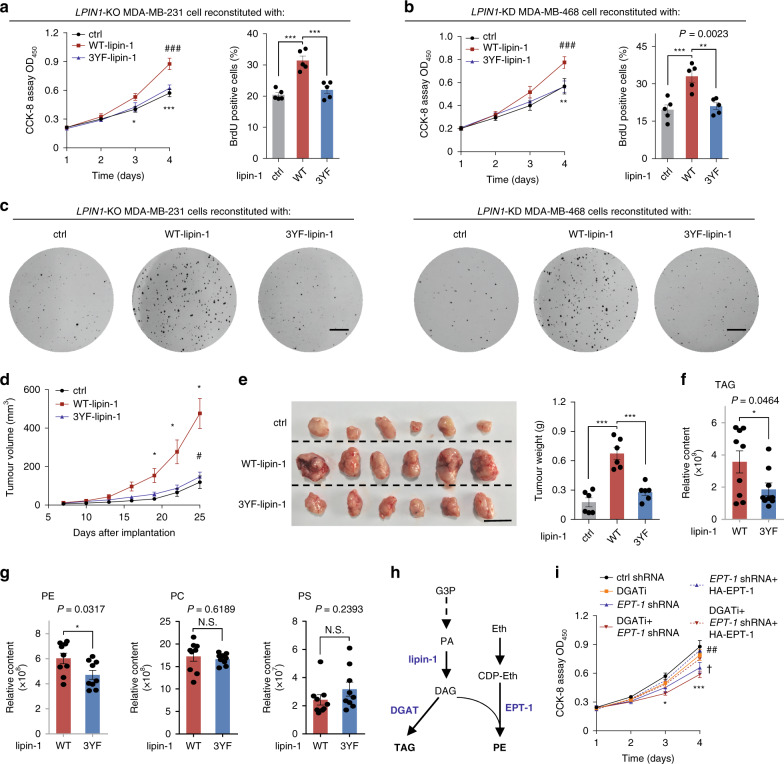
The proliferation of breast cancer cells depends on the tyrosine phosphorylation of lipin-1. **a**, **b** Re-expression of WT-lipin-1 but not 3YF-lipin-1 restored the proliferation of breast cancer cells. *LPIN1-*KO MDA-MB-231 cells (**a**) or MDA-MB-468 cells knocked down of *LPIN1*(*LPIN1*-KD) (**b**) infected with empty vector (ctrl), WT-lipin-1 or 3YF-lipin-1 and were maintained in complete medium containing 10% FBS. The CCK-8 assay (*n* = 4 experiments) and BrdU incorporation assay (*n* = 5 experiments) were performed to determine viable cell number. (Left graph of **a**) ctrl versus WT-lipin-1, *P = 0.0168 (day 3), ***P < 0.001 (day 4); WT-lipin-1 versus 3YF-lipin-1, ^###^*P* < 0.001 (day 4). (Left graph of **b**) ctrl versus WT-lipin-1, **P = 0.0011 (day 4); WT-lipin-1 versus 3YF-lipin-1, ^###^*P* < 0.001 (day 4). **c** The soft agar colony formation assay was performed with *LPIN1*-KO MDA-MB-231 cells or *LPIN1*-KD MDA-MB-468 cells reconstituted with WT-lipin-1, 3YF-lipin-1 or empty vector as a control. The cells were maintained in complete medium containing 10% FBS. Scale bars, 5 mm. **d** Xenograft tumour growth in mice. Volumes of tumour burdened in nude mice receiving *LPIN1*-KO MDA-MB-231 cells reconstituted with WT-lipin-1, 3YF-lipin-1 or empty vector as a control were measured on different days after implantation. *n* = 6 mice per group. Ctrl versus WT-lipin-1, *P = 0.0461 (day 19), *P = 0.0389 (day 22), *P = 0.0102 (day 25); WT-lipin-1 versus 3YF-lipin-1, ^#^*P* = 0.0161 (day 25). **e** Representative images and tumour weights of mouse xenograft tumours from **d**. Scale bar, 10 mm. *n* = 6 mice per group. **f**, **g** Relative levels of TAG (**f**) and phospholipid (**g**) of xenograft tumours from nude mice implanted with *LPIN1*-KO MDA-MB-231 cells reconstituted with WT-lipin-1 or 3YF-lipin-1. *n* = 9 mice per group. The whole list of lipids identified by lipidomics can be found in Supplementary Data 1. **h** Schematic diagram of the synthesis of PE and TAG from glycerol-3-phosphate (G-3-P) in mammalian cells. **i** Knockdown of *EPT-1* or inhibition of TAG synthesis impeded the proliferation of breast cancer cells. MDA-MB-231 cells were knocked down of ethanolamine phosphotransferase-1 (*EPT-1* KD) and/or treated with the DGAT inhibitors PF-04620110 (DGAT1 inhibitor) and PF-06424439 (DGAT2 inhibitor). The *EPT-1* KD MDA-MB-231 cells were infected with HA-EPT-1, and were maintained in complete medium containing 10% FBS. The CCK-8 assays were performed to determine viable cell numbers. Ctrl shRNA versus DGATi + *EPT-1* shRNA, *P = 0.0236 (day 3), ***P < 0.001 (day 4); ctrl shRNA versus *EPT-1* shRNA, ^##^*P* = 0.0027 (day 4); *EPT-1* shRNA versus *EPT-1* shRNA + HA-EPT-1, ^†^*P* = 0.0208 (day 4). **a**, **b**, **i** Quantified in each independent experiment. **d**–**g** Quantified for each xenograft tumour. Data are mean ± s.e.m.; ordinary two-way ANOVA followed by Tukey in (left graph of **a** and **b**, **i**); ordinary one-way ANOVA followed by Tukey in (right graphs of **a** and **b**, **e**); two-way ANOVA (repeated measure) with Geisser–Greenhouse’s correction, followed by Tukey in **d**; two-tailed unpaired Student’s *t*-test in **f**, **g**. ***P < 0.001, N.S. not significant. Source data are provided as a Source Data file.

To examine whether Src-accelerated glycerolipid synthesis affects cell proliferation, we first knocked down *LPIN1* or *SRC* in MDA-MB-231 and MDA-MB-468 cells. It was found that reduction of lipin-1 or Src significantly impeded cell proliferation to similar extents, as indicated by CCK-8 and BrdU assays (Supplementary Fig. 8a, b). Anchorage-independent growth of both cell lines was also found to be similarly dependent on Src and lipin-1 (Supplementary Fig. 8c, d). Importantly, the proliferation retardation caused by knockout of *LPIN1* could be fully rescued by re-expression of WT-lipin-1 but not 3YF-lipin-1 (Fig. 3a–c and Supplementary Fig. 8e–h). Moreover, depletion of lipin-1 significantly retarded tumour growth of the orthotopic xenografts (Supplementary Fig. 8i–l). The xenografts that expressed 3YF-lipin-1 grew significantly slower than WT-lipin-1-expressing tumours (Fig. 3d, e and Supplementary Fig. 8m–r). Untargeted lipidomic profiling on xenograft tumour samples revealed that the levels of PA were remarkably increased, while the levels of DAG were significantly decreased in nude mice implanted with *LPIN1*-KO MDA-MB-231 cells reconstituted with WT-lipin-1 or 3YF-lipin-1 (Supplementary Fig. 8s, t and Supplementary Data 1). More importantly, 3YF-lipin-1 strongly impeded the accumulation of phospholipid PE and moderately reduced TAG levels, while the synthesis of other phospholipids had little changes (Fig. 3f, g, Supplementary Fig. 8u and Supplementary Data 1). To ascertain whether it is the increase of PE or TAG that mediates Src-dependent cell growth, breast cancer cells were knocked down of ethanolamine phosphotransferase-1 (*EPT-1*) to reduce PE biosynthesis (Fig. 3h and Supplementary Fig. 8v), and/or treated with the DGAT inhibitors PF-04620110 (DGAT1 inhibitor) and PF-06424439 (DGAT2 inhibitor), to inhibit TAG biosynthesis (Supplementary Fig. 8w). It was found that while inhibition of TAG biosynthesis showed moderate effects, depletion of EPT-1 impeded the proliferation of MDA-MB-231 cells, which was reversed by lentivirus-mediated re-introduction of EPT-1 (Fig. 3i). Combination of knockdown of *EPT-1* and treatment with DGAT inhibitors impeded cell proliferation more effectively than either EPT-1-downregulation or DGAT inhibition alone (Fig. 3i). Thus, these results indicated that Src-mediated lipin-1 phosphorylation contributes to maintaining the proliferation of breast cancer cells by upregulating phospholipid and TAG synthesis.

### Tyrosine phosphorylation of lipin-1 in mouse mammary carcinoma

**Fig. 4:**
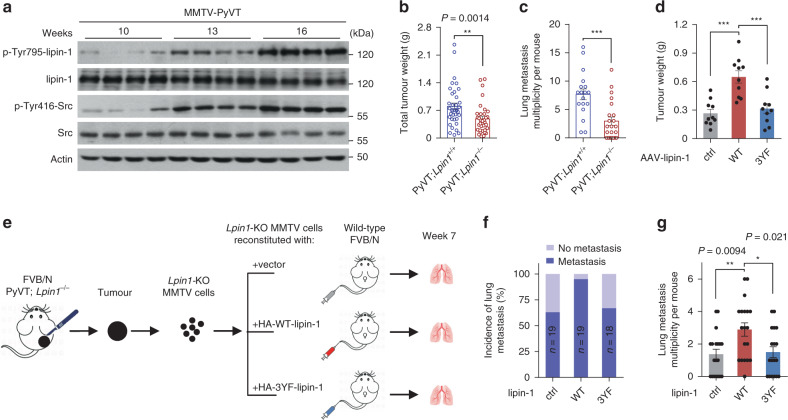
Src-mediated lipin-1 phosphorylation is crucial for breast cancer progression in mice. **a** Immunoblots of breast tumour extract from MMTV-PyVT mice at 10, 13 and 16 weeks. Each lane represents proteins from one mouse. **b** Tumour weights of PyVT;*Lpin1*^+/+^ (*n* = 37) and PyVT;*Lpin1*^*−/−*^ (*n* = 34) mice. Experimental mice were killed at 14 weeks of age, and mammary tumours were then removed and weighed. *n* = 34–37 mice per group. **c** Multiplicity of lung metastasis. Metastasis multiplicity was determined by H&E staining of 14-week-old PyVT;*Lpin1*^+/+^ (*n* = 18) and PyVT;*Lpin1*^*−/−*^ (*n* = 22) mice. **d** Tumour weights of PyVT;*Lpin1*^*−/−*^ mice infected with adeno-associated viruses (AAV) carrying vector (ctrl), WT-lipin-1 or 3YF-lipin-1. The 6-week-old female *Lpin1*^*−/−*^ mice were anaesthetised and injected with 1 × 10^11^ genome copies of AAV per fat pad, at 6 weeks post-injection, tumour weights of these mice were measured. *n* = 10 mice per group. **e** Schematic diagram of intravenous injection to generate experimental lung metastasis. *Lpin1-*KO MMTV cell line was isolated from a spontaneous mammary tumour in PyVT;*Lpin1*^*−/−*^ mice. 1 × 10^5^ *Lpin1-*KO MMTV cells expressing GFP (ctrl), WT-lipin-1 or 3YF-lipin-1 were injected intravenously into 6-week-old FVB/N female mice (*n* = 19 mice for ctrl or WT-lipin-1 group, *n* = 18 mice for 3YF-lipin-1 group). **f** Incidence of lung metastasis. Bars are the percentages of mice with lung metastasis. **g** Lung metastasis multiplicity was calculated at endpoint. **b**–**d**, **g** Data are mean ± s.e.m.; two-tailed Mann–Whitney test in **b**, **c**; one-way ANOVA (repeated measure), followed by Tukey in **d**; ordinary one-way ANOVA, followed by Tukey in **g**. ***P < 0.001, N.S. not significant. Source data are provided as a Source Data file.

We next employed the mice expressing the Polyoma Virus middle T antigen under the direction of the mouse mammary tumour virus promoter/enhancer (MMTV-PyVT) to investigate the effect of lipin-1 in tumour development. These mice have been reported to show Src hyperactivity^26,27^. Along with the development of breast cancer, levels of Tyr-phosphorylated lipin-1 were increased in mammary tumours from the transgenic mice, congruent with elevated levels of the activated p-Tyr416-Src (Fig. 4a). To study for whether lipin-1 would affect the spontaneous development of mammary tumour, we crossed the MMTV-PyVT transgenic mice with *Lpin1*^+/−^ mice, generating PyVT;*Lpin1*^*−/−*^ mice. Deletion of *Lpin1* had no effect on mammary gland morphogenesis as determined by carmine alum staining (Supplementary Fig. 9a). Meanwhile, the tumour frequency or tumour onset was also not significantly affected by lipin-1 deficiency (Supplementary Fig. 9b–d). However, PyVT;*Lpin1*^*−/−*^ mice exhibited an obvious extension of overall survival time (Supplementary Fig. 9e–g). At 14 weeks of age, total tumour weights were significantly reduced in PyVT;*Lpin1*^*−/−*^ mice, along with decreased actively proliferating cells, as indicated by Ki-67 staining (Fig. 4b and Supplementary Fig. 9h, i). Moreover, compared to PyVT;*Lpin1*^*+/+*^ mice, much-increased numbers of the PyVT;*Lpin1*^*−/−*^ mice either were free of, or contained fewer, tumour nodules metastasised to the lung surface (Fig. 4c and Supplementary Fig. 9j, k). However, it is formally possible that germline deletion of lipin-1 could also affect non-tumour cells, which in turn alter tumour metastasis. To clarify this, we transplanted WT and *Lpin1*-KO MMTV tumour cells separately into *Lpin1*^*+/+*^ or *Lpin1*^*−/−*^ mice. While WT tumour cells exhibited stronger metastatic potential in *Lpin1*^*+/+*^ mice, *Lpin1*-KO tumour cells showed equally low metastasis in either *Lpin1*^*+/+*^ or *Lpin1*^*−/−*^ mice (Supplementary Fig. 9l). Analysis of variance (ANOVA) reveals that tumour microenvironment that is determined by host genotypes accounts for 8.89% of the total variance in metastasis, while tumour cell-intrinsic difference that is determined by tumour genotypes accounts for 23.58% of the total variance in metastasis (Supplementary Fig. 9l). These data indicate that although lipin-1 has both cell-autonomous and microenvironment effects, with the former one playing a major part in determining tumour malignancy.

Given that the PyVT tumour model is known to mimic the Luminal B type breast cancer, we also tested the role of lipin-1 in different subtypes of breast cancer patient-derived xenografts (PDXs, Supplementary Data 2). It was found that lipin-1 knockdown in the PDX cells of Luminal A (ER^+^ HER2^−^), HER2^+^ and triple-negative breast cancer (TNBC) rendered these cells less metastatic after tail vein injection into the immunocompromised NOD-SCID female mice (Supplementary Fig. 9m–o), indicating that lipin-1 exerts a general pro-malignancy effect among different breast cancer subtypes.

To investigate the effect of tyrosine phosphorylation of lipin-1 on mouse mammary tumour, we first generated MMTV-Long Terminal Repeat (MMTV/LTR) promoter-driven adeno-associated viruses (AAV) carrying WT-lipin-1, 3YF-lipin-1 or GFP as a control, referred to as AAV-WT-lipin-1, AAV-3YF-lipin-1 and AAV-ctrl. The mammary tissues of PyVT;*Lpin1*^*−/−*^ mice were transduced with these AAVs before the onset of the tumour. It was found that the tumours were heavier in mice injected with AAV-WT-lipin-1 mice compared to AAV-3YF-lipin-1 or AAV-control mice, although tumour onsets were similar among these groups (Fig. 4d and Supplementary Fig. 9p, q). We next isolated and engineered PyVT;*Lpin1*^*−/−*^ mice-derived spontaneous mammary tumour cells with or without lentivirus-mediated re-expression of WT-lipin-1 or 3YF-lipin-1. These cells were then employed in an experimental lung metastasis colonisation experiment by tail vein injection (Fig. 4e). Mice injected with tumour cells expressing 3YF-lipin-1 showed a much lower incidence of lung metastasis and reduced lung seeding compared to mice injected with WT-lipin-1-expressing or control tumour cells (Fig. 4f, g). These data indicated that tyrosine phosphorylation of lipin-1 is crucial for promoting the progression of mouse mammary tumour to malignancy.

### Activation of the Src–lipin-1 axis and human breast cancer malignancy

**Fig. 5:**
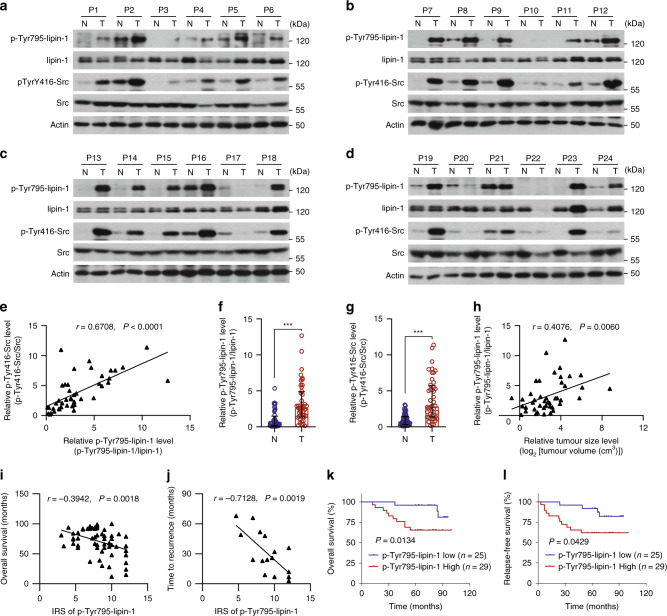
Activation of Src–lipin-1 axis in human breast cancers and its correlation with clinical and pathological parameters. **a**–**d** Immunoblot analysis of p-Tyr795-lipin-1 and p-Tyr416-Src in breast tumour specimens and matched adjacent normal tissues. N tumour-adjacent normal tissue, T tumour, P1 patient 1. **e** The correlation between p-Tyr795-lipin-1 and p-Tyr416-Src in breast cancer. Ratios of p-Tyr795-lipin-1 to total lipin-1 and that of p-Tyr416-Src to total Src were calculated. **f**, **g** Increased p-Tyr795-lipin-1(**f**) and p-Tyr416-Src (**g**) in human breast tumours compared to tumour-adjacent normal tissue. Ratios of phosphorylated protein to total protein were calculated. **h** The correlation between the tumour size and p-Tyr795-lipin-1 in breast cancer patients. Ratios of p-Tyr795-lipin-1 to total lipin-1 was calculated. **i**, **j** The correlation between overall survival (**i**) or time to recurrence (**j**) and p-Tyr795-lipin-1 in breast cancer patients. P-Tyr795-lipin-1 level was assessed by immunoreactive score (IRS) of immunohistochemistry. **k**, **l** Kaplan–Meier survival curve showing overall survival or relapse-free survival of breast cancer patients is reduced in p-Tyr795-lipin-1-high group. P-Tyr795-lipin-1 level was assessed by IRS of immunohistochemistry and separated into higher half and lower half by median. **f**, **g** Data are median with interquartile range, two-tailed Mann–Whitney test; **e**, **h**, **i**, **j** Pearson’s correlation; **k**, **l** analysed using log-rank (Mantel–Cox) test. ***P < 0.001. Source data are provided as a Source Data file.

We next analysed tyrosine phosphorylation of Src and lipin-1 in 44 human primary breast tumours that were instantaneously processed after surgery (Supplementary Data 3). A strong positive correlation between the signal of p-Tyr795-lipin-1 (phosphorylation of lipin-1 on Tyr^795^) and p-Tyr416-Src was revealed (Fig. 5a–e and Supplementary Fig. 10a, b). P-Tyr795-lipin-1 and p-Tyr416-Src were markedly increased in human breast tumours compared to adjacent normal tissues (Fig. 5f, g). Importantly, p-Tyr795-lipin-1 was positively associated with tumour volume, with a significant linear trend identified across the levels of lymph-node metastasis and the clinical stages of the patients (Fig. 5h and Supplementary Fig. 10c, d). Moreover, we evaluated the correlation of p-Tyr795-lipin-1 with patient survival in a cohort of specimens from another 60 patients with invasive breast cancers for which the information of overall survival and relapse-free survival were available (Supplementary Data 4). Significant negative correlations of p-Tyr795-lipin-1 levels with overall survival and relapse-free survival were identified (Fig. 5i, j). A reduction of about 20 months in the median survival period was found in p-Tyr795-high patients, comparing with those of low levels (Fig. 5k and Supplementary Fig. 10e, f). Strikingly, among patients who experienced cancer recurrence, the median time to recurrence of p-Tyr795-high patients was shortened to about 1/6 (upper quartile vs. lower quartile) or 1/3 (higher half vs. lower half) of the p-Tyr795-low patients (Fig. 5l and Supplementary Fig. 10g, h). These clinical associations demonstrate that the Src-mediated lipin-1 tyrosine phosphorylation is highly correlated with human breast cancer malignancy and poorer prognosis.

## Discussion

Src is activated by a variety of extrinsic signals or by mutations in itself and its upstream regulators such as EGFR^28^. It has been reported that lipid droplets are positively correlated with Src levels in a panel of cancer cells^29^. It has become increasingly clear that obesity influences the incidence and the mortality of a large variety of malignancies^30–32^. Obese females were estimated to have more than 50% higher risk to develop breast cancer compared to those with normal BMI^33^. Obese people tend to be associated with larger tumour size, increased lymph-node positivity, and lower survival^29,33,34^. The association between obesity and cancer risk is particularly relevant to breast cancers as they are surrounded by adipose tissue^35,36^. Excess adipose tissue is accompanied by a variety of local changes including altered lipid metabolism, hormone levels, inflammation, which form the microenvironment, as well as systemic changes that alter the physiology^35,37^. Importantly, it was estimated that 40-70% of genetic factors that are related to cancer development were found to be strongly associated with obesity, including those involved in lipid metabolism, such as *LPIN1*, hormone-sensitive lipase *LIPE* and fatty acid synthase *FASN*^12,38,39^.

Because HER2 can decrease the rate of EGF dissociation from EGFR, leading to stronger and prolonged activation of EGFR, we consider EGF as a factor resembling the HER2 branch of Src-activation. By contrast, insulin^40^ and leptin^41^ that also activate Src are closely associated with obesity microenvironment (Supplementary Fig. 10i). Different from EGF, the effects of insulin and leptin in Src-activation are not apparently associated with HER2. Insulin stimulates Src as does EGF, in that activated insulin receptor stabilises the activation loop of Src-family tyrosine kinases^42,43^. Leptin can also stimulate Src and subsequently lipin-1, consistent with the report that experimental hyperleptinemia is associated with Src-activation^44,45^. In addition, inflammatory adipokines such as TNF-α and IL-6 have also been implicated in activating the Src-family kinases via toll-like receptors in various cell types^46^. Moreover, the richness of fatty acids per se that are generally elevated in obesity in the microenvironment may contribute to tumour development^35,37^. Fatty acid-binding proteins (FABPs) that function to solubilise various FAs and coordinate their trafficking inside the cells^47,48^ are recently reported as a new linkage between obesity and breast cancer^49–52^. After entry into cancer cells, exogenous fatty acids cannot only serve as efficient energy-producing nutrients but also act as secondary signalling messengers to coordinate signal transduction cascades that modulate carcinogenic processes^6^. For example, saturated fatty acids alter the membrane distribution of Src, causing it to partition into intracellular membrane subdomains, where it becomes activated^53^. Increased lipogenesis mediated by Src may be particularly relevant to breast cancer. According to Catalogue of Somatic Mutations in Cancer (COSMIC), *Src* is upregulated in 18.84% breast cancer cases, the highest upregulation rate among all Src-family members^54^. Although point mutations and gene amplification of Src alone are not frequent, the total alteration rates among all the Src-family members combined (most of which are cases of gene amplification) are detected in 13% of the breast cancer patients, whose overall survival is shorter than patients without these changes (cBioportal for Cancer Genomics). In line with these data, we found that the activating phosphorylation of Src and Src-mediated tyrosine phosphorylation of lipin-1 are markedly increased in human breast tumours compared to adjacent normal tissues. Moreover, we also identified a strong correlation of the activation of the Src–lipin-1 axis with tumour malignancy and poorer prognosis. Of note, we found that unlike Src, Yes1 does not phosphorylate lipin-1, suggesting that *Src*-family members were not functionally redundant, which is consistent with a previous study showing different effects of c-Src- or c-Yes-deficiency on mouse mammary tumour^27^.

In this study, we demonstrate the tumour intrinsic effect of lipin-1 in causing breast cancer malignancy through promoting lipid synthesis. However, one question remains in this study is whether lipin-1 in non-tumour cells also functions to shape pro-metastatic microenvironment. We show that the metastatic potential of WT MMTV tumour cells is reduced in *Lpin1*^*−/−*^ mice compared to *Lpin1*^*+/+*^ mice, and the non-tumour effect of lipin-1 contributes to 8.89% of the total variance in metastasis. It is conceivable that the presence or absence of lipin-1 in the neighbouring non-tumour cells would give rise to different compositions that would, in turn, alter contact between cancer and non-cancer cells or the contact between cancer cells and extracellular matrix.

In sum, it is remarkable that the proto-oncogene *Src*, apart from stimulating pro-mitogenic signalling cascades to cause tumours, plays a direct role in reprogrammed lipid metabolism, which endows cells with advantages in proliferation and metastasis.

## Methods

### Generation of CRISPR/Cas9-mediated knockout cell lines

Human codon-optimised Cas9 (hCas9) and GFP-targeting gRNA-expressing plasmids were purchased from Addgene. The GFP-targeting sequence in the gRNA vector was replaced with the sequence 5′-AATTACGTGGGGCAGTTAGC-3′ targeting exon 3 of human *LPIN1* or 5′-CGCCGCTGGCTGGCATCCTT-3′ targeting exon 4 of human *SRC*. To construct the knockout cell lines, MDA-MB-231 cells were transfected with 1.5 μg of gRNA-expressing plasmid and 1.5 μg of hCas9 plasmid and subjected to blasticidin selection for 3 days. The resultant resistant cells were then sorted into single clones in the 96-well plate. Single clones were screened by genomic PCR with primers targeting the upstream region of the gRNA (5′-CCGGAAATGAGAGGAGCTGA-3′ for human *LPIN1* or 5′-GTGTCTTCTCTCTCTCCTG CCA-3′ for human *SRC*) and the downstream region of the gRNA (5′-CAAGACAGGTGGCATCACCA-3′ for human *LPIN1* or 5′-GAGTTGAAGCCTCCGAA CAG-3′ for human *SRC*). Positive clones were further verified by immunoblotting. The sequences of the primers are also shown in Supplementary Data 5.

### Protein analysis by mass spectrometry

To identify the lipin-1 interacting proteins, Flag-tagged lipin-1 was expressed in *LPIN1*-KO MDA-MB-231 cells, immunoprecipitated with antibody to Flag, and subjected to SDS-PAGE, followed by silver staining. Gels were excised and digested by trypsin, followed by mass spectrometry analysis. To identify the tyrosine phosphorylation sites on lipin-1, HEK293T cells were transfected with Flag-lipin-1 with HA-Src or empty vector as a control. Flag-lipin-1 was then immunoprecipitated with anti-Flag antibody and subjected to SDS-PAGE, followed by Coomassie blue staining. The bands corresponding to lipin-1 were excised and digested by trypsin, followed by mass spectrometry analysis.

### Antibodies and reagents

Antibodies for phospho-Ser^106^-lipin-1 (1:1000 for IB) were described previously^18^. The rabbit polyclonal antibodies that specifically recognise phosphorylated lipin-1 were generated by immunising rabbits with synthetic peptides as follows: DKRSRHLGADGV(pTyr)LD for phospho-Tyr^398^-lipin-1 (1:500 for IB), EVAAL(pTyr)FPKNGDPSG for phospho-Tyr^413^-lipin-1 (1:500 for IB), and TEPF(pTyr)AAFGNRPADV for phospho-Tyr^795^-lipin-1 (1:500 for IB, 1:50 for IHC). Antibodies to phospho-Tyr^416^-Src (1:1000, Cat. 6943), Src (1:1000, Cat. 2123), lipin-1 (Cat. 14906), phospho-Tyrosine (1:1000, Cat. 9411), β-tubulin (1:1000, Cat. 2128), phospho-p70 S6 Kinase (1:1000, Cat. 9205), S70 S6 Kinase (1:1000, Cat. 9202) and Ki-67 (1:400 for IHC, Cat. 12202) were purchased from Cell Signaling Technology. Antibodies to lipin-1 (1:1000 for IB, H-120; 1:50 for IF, B-12), HA (1:1000, Y11, F7), normal Rabbit IgG (1:5000, sc-2027) and c-Src (1:1000, B12) were obtained from Santa Cruz Biotechnology. Antibodies to Flag (1:5000, Cat. F2555 and SAB4200071) and Actin (1:5000, Cat. A1978) were purchased from Sigma. Anti-Calnexin (1:1000 for IB, 1:100 for IF, Cat. 10427-2-AP) and anti-GST (1:5000, Cat. 66001-2-Ig) antibodies were purchased from Proteintech. Anti-LPIN3 (1:500, Cat. LS-C339442) was from LSBio. Anti-SELI (1:1000, EPT-1, Cat. H00085465-A01) was from Novus Biologicals. Donkey anti-rabbit IgG secondary antibody Alexa-Fluor 488 (Diluted 1:100 in PBS, Cat. A21206), and donkey anti-mouse IgG secondary antibody Alexa-Fluor 594 (Diluted 1:100 in PBS, Cat. A21203) were purchased from ThermoFisher Scientific. The HRP-conjugated goat anti-Mouse IgG (Cat. 115-035-003, 1:5000 for IB) and HRP-conjugated goat anti-Rabbit IgG (Cat. 111-035-003, 1:5000 for IB) antibodies were purchased from Jackson ImmunoResearch. Anti-Flag beads (M_2_) (Cat. M8823) and oleic acid (Cat. O1383) were purchased from Sigma. Rapamycin (Cat. S1039), Torin 1 (Cat. S2827), Blasticidin (Cat. S7419), Src inhibitors Dasatinib (Cat. S1021) and SKI-606 (Cat. S1014) were purchased from Selleckchem. Recombinant human EGF (Cat. 236-EG), human PDGF-BB (Cat. 220-BB) and human IGF-1 (Cat. 291-G1) proteins were purchased from R&D Systems. [9,10-^3^H(N)]-Oleic Acid (Cat. NET289001MC) and [γ-^32^P]-ATP (Cat. NEG502Z250UC) were purchased from PerkinElmer. l-a-dipalmitoyl [2-palmitoyl-1-^14^C]-Phosphatidic Acid (Cat. ARC 1574) was obtained from American Radiolabeled Chemicals.

### Plasmid construction

Mouse Src was kindly provided by Q. Li (Xiamen University, Fujian, China). Point mutations of Src and lipin-1 were performed by a PCR based site-directed mutagenesis method using PrimeSTAR polymerase (Takara). Full-length cDNAs encoding human GK, GPAM, AGPAT1, MGAT1, MGAT2, DGAT1, DGAT2, Src, Yes1, Fyn and Lyn were obtained by PCR using human cDNA generated from HEK293T cells. The coding sequences for the various proteins were cloned into pcDNA3.3 vector for transfection, into pBOBI vector for lentiviral infection, or into pAAV vector containing Mouse Mammary Tumour Virus Long Terminal Repeat (MMTV-LTR) promoter for adeno-associated virus (AAV) infection. All PCR products were verified by sequencing. Primer sequences used for these constructs are available upon request.

### Cell culture and transfection

SK-BR3 and MCF-7 cells were provided by X. Li (Xiamen University, Fujian, China). HEK293T, MDA-MB-231 and MDA-MB-468 were purchased from ATCC. MMTV mouse mammary tumour cell line, established from a tumour of MMTV-PyVT mouse, was provided by G. Ouyang (Xiamen University, Fujian, China). All cell lines were maintained at 37 °C and 5% CO_2_, with the exception of MDA-MB-231 and MDA-MB-468 cells, which were cultured without CO_2_. HEK293T and MMTV cells were cultured in Dulbecco’s modified Eagle’s medium (DMEM) supplemented with 10% fetal bovine serum (FBS) and 100 IU penicillin and 100 mg/ml streptomycin (P/S). SK-BR3 cells were maintained in McCoy’s 5a medium (Gibco) supplemented with 10% FBS and P/S. MDA-MB-231 and MDA-MB-468 cells were kept in L-15 medium (Gibco) supplemented with 10% FBS and P/S. All cell lines were validated to be free of mycoplasma contamination. Polyethylenimine (Polyscience, Cat. 23966) at a final concentration of 10 μM was used for transfection of HEK293T cells. Cells were harvested 20–24 h after transfection with a lysis buffer [20 mM Tris-HCl (pH 7.4), 150 mM NaCl, 1 mM EDTA, 1 mM EGTA, 1% Triton, 2.5 mM sodium pyrophosphate, 1 mM β-glycerolphosphate, 1 mM Na_3_VO_4_, 2 μg/ml leupeptin and 1 mM PMSF] and subjected to immunoprecipitation.

### RNA interference and lentivirus-mediated infection

The lentivirus-based vector pLL3.7 was used for expression of shRNAs in cells. The 19-nucleotide sequence for each shRNA is as follows: 5′-GCTCCAGATTGTCAACAAC-3′ (#1) and 5′-GGACCTTCCTCGTGCGAGA-3′ (#2) for human *SRC*, 5′-GCATTGACATCATTGTCAT-3′ for human *LPIN1*, 5′-GGAGACAACGGAGAAGCAT-3′ for mouse *Lpin1*, 5′-GCGTGTCCTAAGAGTGATT-3′ for human *LPIN2*, 5′-GCCTCTCCTCCGATCAGAT-3′ for human *LPIN3*, 5′-GCTTTCTGCTGGTCGTATT-3′ for human *EPT-1*. pLL3.7-*Renilla* was used to express control shRNA (5′-GTAGCGCGGTGTATTATAC-3′). The sequences of the primers are also shown in Supplementary Data 5. Lentiviruses for infection were packaged in HEK293T cells using a liposomal transfection reagent (YEASEN, Cat. 40802ES03). Viruses were collected 48–72 h after transfection and stored at −80 °C until use. MDA-MB-231, MDA-MB-468 or MMTV cells were cultured in virus-containing medium supplemented with 10 μg/ml polybrene for 24 h. The dishes containing cells and viruses were then centrifuged at 1000 × *g* for 30 min. At 12 h after infection, the medium was refreshed. All experiments were carried out at least 48 h after infection.

### Immunoprecipitation and immunoblotting

Cells for immunoprecipitation were lysed on ice using lysis buffer [20 mM Tris-HCl (pH 7.4), 150 mM NaCl, 1 mM EDTA, 1 mM EGTA, 1% Triton, 2.5 mM sodium pyrophosphate, 1 mM β-glycerolphosphate, 1 mM Na_3_VO_4_, 2 μg/ml leupeptin and 1 mM PMSF], and subjected to immunoprecipitation using antibodies as indicated. The immunoprecipitates were then washed three times with lysis buffer and solubilized in SDS buffer for immunoblotting. The total cell lysates and immunoprecipitates were boiled and separated on 8–15% SDS-PAGE, followed by electrophoretic transfer. Primary antibodies were used according to manufacturers’ protocols. Proteins were visualised by enhanced chemiluminescence using horseradish peroxidase-conjugated antibodies.

### Protein purification and in vitro GST pull-down assay

Full-length cDNAs of lipin-1, non-phosphorylatable mutants of lipin-1 (3YF-lipin-1), Src and a kinase-dead form of Src (KD-Src) were cloned into pET-28a or pGEX 4T-1 vector. Protein expression was performed in the strain *E. coli* BL21 strain. The transformed *E. coli* cells were incubated for 2 h with 1 mM isopropyl-d-thiogalactopyranoside to induce the expression of His-tagged lipin-1, Src and KD-Src or GST-tagged Src. Ni^2+^-NTA-agarose (Sigma, Cat. P6611) was used for the purification of His-tagged proteins and glutathione sepharose beads (GE Healthcare, Cat. 17513202) were used for the purification of GST-tagged Src. For GST pull-down assay, the beads-bound GST or GST-Src proteins were incubated with purified His-lipin-1 in GST pull-down buffer [150 mM NaCl, 50 mM Tris-HCl (pH 7.6), 0.5% Nonidet P-40 (NP-40), and 1 mM PMSF] at 4 °C for 3 h. The precipitates were washed for 3 times, separated by SDS-PAGE and visualised by coomassie brilliant blue staining. Immunoblot was also carried out using anti-lipin-1 and anti-GST antibodies to detect His-lipin-1, GST or GST-Src.

### In vitro kinase assay

In vitro kinase assay was performed based on a previously described protocol^55^. In brief, purified bacterially expressed His-tagged WT-lipin-1 or 3YF-lipin-1 and purified His-tagged WT-Src or KD-Src were incubated in a kinase buffer containing 25 mM HEPES (pH 7.4), 50 mM NaCl, 5 mM MgCl_2_, 1 mM DTT, 0.5 mg/ml BSA, 1 mM Na_3_VO_4_, 30 μM cold ATP and 0.5 μCi ^32^P-labelled ATP (PerkinElmer, Cat. NEG502Z250UC) for 30 min at 28 °C. Reactions were stopped by addition of SDS sample buffer, followed by SDS-PAGE, transferring to PVDF membrane and autoradiography.

### PAP activity measurement

For the PAP activity measurement of ectopically expressed lipin-1, Flag-tagged WT-lipin-1, 3YF-lipin-1, 3YE-lipin-1 and 3YD-lipin-1 were individually transfected with or without HA-tagged Src into HEK293T cells. Cells were lysed 24 h after transfection. Flag antibody-conjugated beads (Sigma, Cat. M8823) were then used for immunoprecipitation of lipin-1 for 3 h at 4 °C. The immunoprecipitates were then washed three times with lysis buffer and twice with 25 mM Tris-HCl (pH 7.5) buffer. The Flag-tagged proteins were then eluted with 3 × Flag peptide (Sigma, Cat. F3290) in 25 mM Tris-HCl (pH 7.5) buffer containing 150 mM NaCl and protease inhibitors. For measurement of the PAP activity of endogenous lipin-1, MDA-MB-231 or MDA-MB-468 cells were seeded in 100-mm dishes and grown in complete L-15 medium for 12–16 h. Then, cells were incubated with L-15 medium with or without serum for 4 h and then treated with or without serum or EGF. Cells were then washed once with ice-cold PBS, harvested with Triton X-100 lysis buffer and followed by determination of PAP activity.

The PAP assay was carried out based on a previously described protocol^18^. The PAP activity was measured in a reaction buffer [0.1 M Tris/maleate (pH 6.9), 10 mM MgCl_2_, 0.2% fatty acid-free BSA, 1 mM DTT, and 1 × EDTA-free protease inhibitor with or without 12.5 mM *N*-ethylmaleimide (Sigma, Cat. E3876)] supplemented with 5 μl of [^14^C] PA mixture for 20 min at 37 °C. The [^14^C] PA mixture used was prepared as follows: 15 μl 0.1 mCi/ml of the [^14^C]PA (American Radiolabeled Chemicals, Cat. ARC 1574) was mixed with 3 mM 3-sn-PA (Sigma, Cat. P9511) and 2 mM PC (Sigma, Cat. P3556) in 1 ml of chloroform, evaporated under a stream of N_2_, and resuspended in 1 ml of ice-cold distilled deionized water. This mixture was sonicated 10 s for three times on the ice. The reaction was terminated by adding 1 ml of a chloroform-methanol mixture (19:1, v/v) containing 0.8% olive oil to each tube with vortexing. 500 mg of activated Al_2_O_3_ was then added to each tube. After three cycles of 30 s vortexing, samples were centrifuged at 10,000×*g* for 10 min, and 350 μl of supernatants were used for scintillation counting of the ^14^C radioactivity. PAP activities were normalised to the protein concentration present in the starting lysate determined by Bradford assay.

### Subcellular fractionation

The subcellular fractionation was carried out based on a previously described protocol^17^. MDA-MB-231 cells were rinsed in ice-cold PBS once and resuspended in 1 ml of separation buffer containing 50 mM Tris-HCl (pH 7.4), 0.25 M sucrose, 50 mM NaF, 1 mM EDTA, 1 mM PMSF, 2 μg/ml leupeptin and 1 mM Na_3_VO_4_. The cells were then mechanically broken by passing through a 25 gauge needle for 14 times and subjected to centrifugation at 100 × *g* for 10 min, 16,000 × *g* for 20 min and 175,000 × *g* for 1 h at 4 °C sequentially to collect the nucleus fraction, plasma membranes and mitochondria fraction, and microsomal fraction in the pellets and cytosol fraction of the final supernatant. The pellets from each centrifugation were washed three times with the separation buffer to eliminate the residual contamination from the corresponding supernatants.

### Glycerolipid synthesis assay

MDA-MB-231 and MDA-MB-468 cells were treated with 0.25 mM BSA-bound oleic acid mixed with 3 μCi [9, 10-^3^H]-oleic acid/ml and cultured for another 1.5 h. Cells were collected and total lipids were extracted and dried. The resuspended lipid extract was separated by thin-layer chromatography (TLC) on silica layers (Macherey-Nagel, TLC Plates Polygram SIL G 805013)^56^. TAG and DAG were separated with hexane:diethylether:acetic acid (80:20:1, v/v/v) as a solvent. Phospholipids (PC, PE, PS and PI) was separated with chloroform:ethanol:water:trimethylamine (30:35:6:35, v/v/v/v). Bands of TAG, DAG and phospholipids indicated by commercial standards were scraped from the plates, followed by scintillation counting.

### Fatty acid oxidation measurement

The fatty acid oxidation rates of MDA-MB-231 and MDA-MB-468 cells were determined based on a previously described protocol^18^. Briefly, cells were cultured in 6-well plates at 37 °C. After 18–24 h, cells were washed once with PBS, and then were incubated in 1 ml of reaction buffer containing 10 mM HEPES (pH 7.4), 120 mM NaCl, 5 mM KCl, 2.6 mM KH_2_PO_4_, 2.6 mM MgSO_4_, 2 mM CaCl_2_, 25 mM NaHCO_3_, 1 mM BSA-bound oleic acid and 0.8 μCi/ml [9,10-^3^H(N)]-oleic acid for 1 h at 37 °C. 480 μl of supernatant was then transferred and mixed with 192 μl of 1.3 M perchloric acid, followed by centrifugation at 10,000 × *g* for 1 min to remove precipitated proteins. 500 μl supernatant was then mixed with 4 ml of scintillation liquid to determine the ^3^H radioactivity. Cell-free samples were also used for each experiment to ensure that no more than 20% of labelled oleic acid was oxidised during the assay. The fatty acids are β-oxidised to produce ^3^H_2_O, the amounts of which were determined by a scintillation counter (DPM for 1 h). Protein concentrations were recorded for each sample by Bradford assay. The amount of ^3^H_2_O generated was expressed as DPM oxidised fatty acid per mg protein per hour.

### Immunofluorescence

Immunofluorescence was performed based on a previously described protocol^57^. In brief, MDA-MB-231 cells were plated on glass coverslips in 12-well plates at 30–40% of confluence. Cells were treated with BSA or OA 2 h before fixation. After fixation in 4% paraformaldehyde, the cells were rinsed with PBS, permeabilized with 0.05% saponin in PBS for 10 min, and then blocked with 5% normal donkey serum for 1 h at room temperature. The coverslips were incubated with antibodies to lipin-1 and Calnexin overnight at 4 °C. Alexa-Fluor 488-conjugated and 594-conjugated secondary antibodies were used to label the target protein. Fluorescence images were captured using a Zeiss Laser Scanning Microscope 780 confocal microscope. Quantification was performed by using the JACoP plugin for Image J.

### Cell proliferation and anchorage-independent growth assay

Cell counting kit-8 (CCK-8) (MCE, Cat. HY-K0301) and cell proliferation ELISA, BrdU (colorimetric) (Roche, Cat. 11647229001) were used to determine cell proliferation according to manufacturer’s instructions. For anchorage-independent growth in soft agar, the bottom layer was generated by covering 6-well plate with 1.5 ml of 0.5% Nobel Agar (BD, Cat. 214220) in a growth medium that was then solidified at 37 °C for 30 min. 5000 cells were plated on the bottom layers in 1.5 ml of 0.3% Nobel Agar in the growth medium. A layer of growth medium was maintained over the upper layer of agar to prevent desiccation. Cells were maintained at 37 °C for 3–4 weeks for colony formation, followed by staining with nitroblue tetrazolium chloride (1 mg/ml) (Sangon Biotech, Cat. A100329-0500). Images were taken by an Epson Expression 11000XL Graphics Arts scanner.

### Animal studies

All animal procedures were performed with an approved protocol from the Institutional Animal Care and Use Committee at Xiamen University. Mice were housed in a temperature-controlled environment under a 12 h light:dark cycle with free access to water and standard rodent chow diet at 23 ± 3 °C and 30–70% humidity. FVB/N-Tg (MMTV-PyVT) 634Mul/J (PyVT, Stock No. 002374) and BALB/cByJ-*Lpin1*^*fld*^/J (*Lpin1*^+/−^, Stock No. 001592) were obtained from Jackson Laboratories. To generate PyVT;*Lpin1*^*−/−*^ mice, *Lpin1*^+/−^ mice on BALB/c background were back-crossed with wild-type mice on FVB/N background for six generations to generate the FVB/N *Lpin1*^+/−^ mice. Female *Lpin1*^*+*/−^ mice were then crossed with male PyVT;*Lpin1*^*+*/−^ mice. PCR genotyping of *Lpin1*^*−/−*^ mice was performed with primers Fld-WT-Fwd: 5′-CCGAGCATTAAAGGATAGGTTG-3′, Fld-Com-Rev: 5′-AGT GCACGCTAAGGAAATGC-3′ and Fld-MUT-Fwd: 5′-TCCTCACCTGATCGTTGTCA-3′, resulting in an amplicon of ~620 bp of knockout allele and 692 bp of the wild-type allele. Genotyping of mice expressing MMTV-PyVT has performed with primers Fwd: 5′-GGAAGCAAGTACTTCACAAGGG-3′ and Rev: 5′-GGAAAGTCACTAGGAGCAGGG-3′, resulting in a 556 bp product. The sequences of the primers are also shown in Supplementary Data 5.

Mice were checked every 3 days for examining sizes of primary tumours and tumour-free survival time. Maximum tumour diameters were not allowed to exceed 20 mm except for the determination of overall survival time. Experimental mice were killed at 10–16 weeks of age, and mammary tumours were then removed and weighed. Mammary tumours and lung tissues were fixed with 4% paraformaldehyde and paraffin-embedded for histology study. Sections of 5 μm were used for haematoxylin and eosin (H&E) staining.

For xenograft studies, cells were washed twice with PBS and concentrated to 1 × 10^6^ per 50 μl in PBS. The cell was then suspended in PBS and mixed with an equal volume of Matrigel (Corning, Cat. 356230). 100 μl of cells mixed with Matrigel were orthotopically injected into the fourth mammary fat pad of 8-week-old female nude mice or FVB/N female mice. One week post-injection, tumour volumes were measured every 3 days (tumour volume = width^2^ × length × 1/2). Mice were then euthanized and xenograft tumours were dissected for analysis. For lung metastasis colonisation model, PyVT;*Lpin1*^*−/−*^ mice-derived tumour cells (1 × 10^5^) re-expressing WT-lipin-1 or 3YF-lipin-1, or without lipin-1 were injected through the tail vein of 6-week-old FVB/N female mice; 1 × 10^5^ WT or *Lpin1-*KO MMTV cells were injected intravenously into either six-week-old *Lpin1*^+/+^ or *Lpin1*^*−/−*^ female mice; the patient-derived xenograft (PDX)-derived Luminal A (ER^+^HER2^−^), triple-negative breast cancer (TNBC) or HER2 positive (HER2^+^) breast cancer cells expressing shRNA targeting *LPIN1* (*LPIN1* shRNA) or *Renilla* as a control (ctrl shRNA) were injected through the tail vein of six-week-old immunocompromised NOD-SCID female mice.

### Carmine alum staining of the mammary fat pad

The fourth mammary fat pads were removed from *Lpin1*^+/+^ and *Lpin1*^*−/−*^ mice and quickly spread onto ice-cold glass slides, and then fixed in Carnoy’s fixative (ethanol:chloroform:glacial acetic acid = 6:3:1, v/v/v) for 2–4 h. The glands were then washed with 70% ethanol for 15 min, rinsed in distilled water once for 5 min, and stained with Carmine Alum (Stem Cell Technologies, Cat. 07070) according to the manufacturer’s instructions. The slides were washed with ethanol (70%, 95% and 100%), cleared with xylene and mounted with neutral balsam. Images were taken an Epson Expression 11000XL Graphics Arts scanner.

### Immunohistochemistry

Sections of 5 μm were baked 4 h at 75 °C, followed by deparaffinization and rehydration with xylene and a graded series of ethanol. The sections were then washed with PBS, boiled in 10 mM sodium citrate buffer (pH 6.0) at 100 °C for 30 min. After rinsing twice in PBS, sections were incubated with 3% H_2_O_2_ for 30 min to block endogenous peroxidase, blocked at room temperature for 1 h by using 1% BSA, followed by incubation with p-Y795-lipin-1 or Ki-67 antibodies overnight at 4 °C. Following PBS wash, sections were then incubated with HRP-conjugated goat anti-rabbit or mouse secondary antibody for 1 h at room temperature. Sections were stained by DAB Peroxidase (HRP) Substrate Kit (Vector Laboratories, Cat. SK-4100) and then counterstained with hematoxylin according to manufacturer’s protocols.

### Generation and delivery of recombinant adeno-associated viruses (AAV)

To generate AAV of serotype 9 (AAV-9), HEK293T cells were transfected with AAV transfer plasmid containing sequence encoding WT-lipin-1, 3YF-lipin-1 or GFP as a control, together with AAV-9 helper plasmids. After 60–72 h of transfection, cells were harvested and resuspended with buffer A (50 mM Tris-HCl (pH 8.0) and 150 mM NaCl). The crude virus was released from the cell suspension after three freeze/thaw cycles and purified with a graded series of Density Gradient Medium (Sigma, Cat. D1556). The virus fraction was dialysed in PBS and concentrated by Millipore Ultra centrifugal filter. AAV titre was determined by RT-PCR using primers targeting the MMTV-LTR promoter. The 6-week-old *Lpin1*^*−/−*^ female mice were anaesthetised and injected with 1 × 10^11^ genome copies of AAV per fat pad, 6 weeks post-injection, tumour weights of these mice were measured.

### Clinical samples

Human breast cancer samples were from Sun Yat-Sen University Cancer Center (Guangzhou, China) with written informed consent from the patients. All the procedures were approved by the Institutional Review Board of Sun Yat-Sen University Cancer Center (Guangzhou, China) and conducted in accordance with the Declaration of Helsinki. Breast tumour samples (44 tumour sample and 44 matching adjacent normal tissues) were obtained from patients and were quickly processed to ensure the quality of the clinical samples, and stored at −80 °C wherever necessary for further use. The clinical stages of breast cancer were classified according to the tumour-node-metastasis classification of the Sixth Edition of the AJCC Cancer Staging Manual.

The breast cancer tissue microarray (60 cases) was prepared at the Xijing Hospital, Fourth Military Medical University, Shaanxi, China. The immunohistochemistry data were quantified using the immunoreactive score (IRS) system^58^. Each sample was evaluated by 3 persons individually in a blinded manner and the mean score was considered as the final IRS. Patients were grouped based on IRS scores of p-Y795-lipin-1 levels. The p-Y795-lipin-1 high- and low- groups were determined based on the median or quartiles calculated across the entire dataset.

### Lipid extraction and lipodomic analysis by LC–MS/MS

Frozen tumour tissues were inactivated by addition of 1 ml mixture containing methanol, MTBE and internal standard mixture. Tissue samples were homogenised on an automated bead ruptor and whirled the mixture at 4 °C for 2 min. Then, 500 µl of deionized H_2_O was added to the mixture, followed by centrifuged with 16,000 × *g* at 4 °C for 10 min. The extract supernatant was transferred to a new tube and dried. Samples were stored at −80 °C until mass spectrometric analysis.

The lipodomic analysis by LC–MS/MS was performed by Metware Biotechnology Co., Ltd (Wuhan, China) based on a previously described protocol^59^, with slight modifications. In brief, the sample extracts were analysed using a LC-ESI-MS/MS system (UPLC, Shim-pack UFLC SHIMADZU CBM A system, https://www.shimadzu.com/; MS, Q TRAP System, https://sciex.com/). The analytical conditions were as follows, UPLC: column [Thermo C30 (2.6 μm, 2.1 mm × 100 mm)], solvent system [A: acetonitrile/water (60/40, v/v) containing 0.04% acetic acid and 5 mM ammonium formate, B: acetonitrile/isopropanol (10/90, v/v) containing 0.04% acetic acid and 5 mM ammonium formate], a graded series of A/B programme [80:20 (v/v) at 0 min, 50:50 (v/v) at 3.0 min, 35:65 (v/v) at 5 min, 25:75 (v/v) at 9 min and 10:90 (v/v) at 15.5 min], flow rate (0.35 ml/min), temperature, 45 °C; injection volume: 2 μl. The effluent was alternatively connected to an ESI-triple quadrupole-linear ion trap (QTRAP)-MS.

LIT and triple quadrupole (QQQ) scans were acquired on a triple quadrupole-linear ion trap mass spectrometer (Q TRAP), Q TRAP LC–MS/MS System, equipped with an ESI Turbo Ion-Spray interface, operating in positive and negative ion mode and controlled by Analyst 1.6.3 software (AB Sciex). The ESI source operation parameters were as follows: an ion source, turbo spray; source temperature 550 °C; ion-spray voltage (IS) 5500 V; ion source gas I (GSI), gas II (GSII), curtain gas (CUR) were set at 55, 60 and 25 psi, respectively; the collision gas (CAD) was medium. Instrument tuning and mass calibration were performed with 10 and 100 μM polypropylene glycol solutions in QQQ and LIT modes, respectively. The QQQ scans were acquired as MRM experiments with collision gas (nitrogen) set to 5 psi. DP and CE for individual MRM transitions were done with further DP and CE optimisation. A specific set of MRM transitions were monitored for each period according to the metabolites eluted within this period.

### Statistics and reproducibility

Data were analysed using GraphPad Prism built-in tests. For experiments with only two groups, two-tailed Student’s *t*-test or Mann–Whitney test was used for statistical comparisons based on the results of the normality test of each group of data. Welch’s correction was used for unequal variances. One-way or two-way ANOVA (ordinary or repeated measure) with post-tests (Tukey, Sidak, Dunnett) as indicated in the figure legends was used for comparison of data with multiple groups. Geisser–Greenhouse’s correction was used for ANOVA tests where applicable. Pearson’s correlation test was used for a measure of the strength of the association between two variables. Log-rank (Mantel–Cox) test was used to compare the survival distributions of the two groups. For other graphs showing representative data, reproducibility is stated below: (1) *n* ≥ 3 biologically independent experiments for Figs. 1a–f; 2a–c, f; 3c; 4a; Supplementary Figs. 1b; 2c–h; 3b–j, l; 4d–f; 5a–f; 6d, e; 7c, g; 8a, b, e, f (immunoblotting), i, k, o, v (immunoblotting); 9m–o (immunoblotting), p. (2) *n* = 3 technically independent experiments for Fig. 5a–d, Supplementary Figs. 10a, b. (3) *n* = 1 experiment for Supplementary Fig. 2a.

### Reporting summary

Further information on research design is available in the Nature Research Reporting Summary linked to this article.

## Supplementary information

Supplementary Information

Description of Additional Supplementary Files

Supplementary Data 1

Supplementary Data 2

Supplementary Data 3

Supplementary Data 4

Supplementary Data 5

Reporting Summary

## Data Availability

The authors declare that all the data supporting the findings of this study are available within the paper and its supplementary information files. Primary antibodies and the nucleotide sequence for each shRNA used in this study are described in the ‘Methods’ section. Source data are provided with this paper.

## Source data

Source Data
