# Supplementary material for: Proto-oncogene Src links lipogenesis via lipin-1 to breast cancer malignancy
Source: Nat Commun. 2020 Nov 17;11:5842. doi: 10.1038/s41467-020-19694-w (PMC7672079; doi:10.1038/s41467-020-19694-w)
Supplement: Supplementary file 1 — Supplementary Information [file 41467_2020_19694_MOESM1_ESM.pdf]

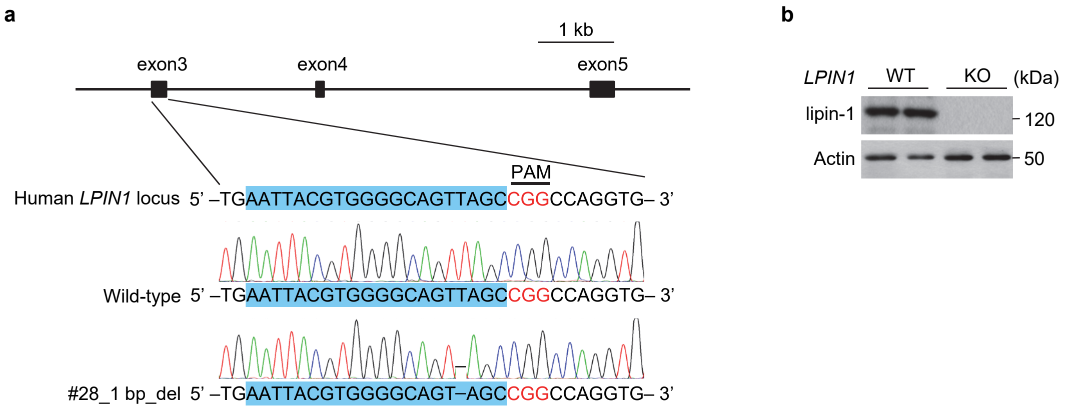

Supplementary Figure 1. Generation and validation of *LPIN1*-knockout cell line.
a, Schematic of CRISPR-Cas9-mediated knockout (KO) of *LPIN1* in MDA-MB-231 cells. The gRNA-targeting sequence used is highlighted in blue and the protospacer-adjacent motif (PAM) sequence is indicated in red. The 1 base pair (bp) deletion of the resulting lipin-1 gene was verified by sequencing (clone #28). **b,** Immunoblot analysis of proteins of CRISPR control (WT) or *LPIN1*-KO MDA-MB-231 cells. Source data are provided as a Source Data file.

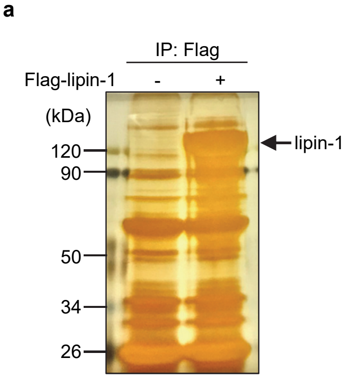

b

lipin-1 co-immunoprecipitates	Peptide (No.)
Proto-oncogene tyrosine-protein kinase (Src)	13
Desmoglein-2 (DSG2)	12
TGF-beta-activated kinase 1(MAP3K7)-binding protein 1 (TAB1)	11
Coatomer protein complex subunit alpha (COPα)	10
Glycogen synthase kinase-3 alpha (GSK3α)	5
Thioredoxin domain-containing protein 5 (TXND5)	5
F-box/WD repeat-containing protein 11 (FBW11)	4
Signal recognition particle subunit SRP68 (SRP68)	4

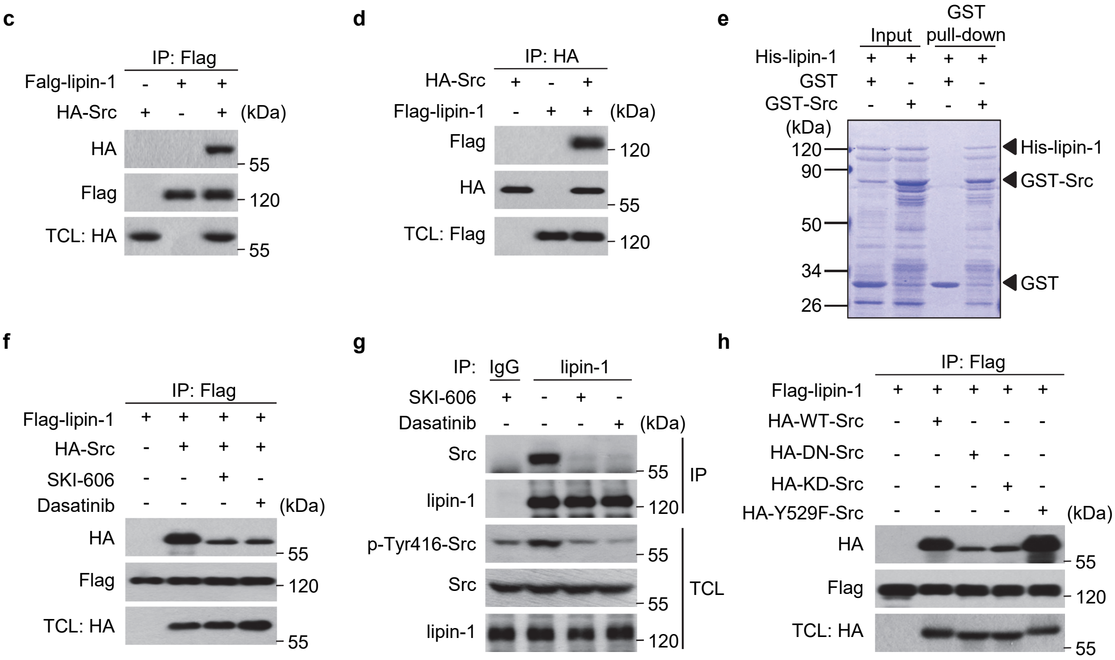

Supplementary Figure 2. Kinase-active conformation of Src is essential for its interaction with lipin-1.
a, Silver staining of lipin-1-interacting proteins. Flag-tagged lipin-1 was expressed in *LPIN1*-KO MDA-MB-231 cells. Cells were maintained in complete medium containing 10% FBS and lysed and subjected to immunoprecipitation (IP) against Flag, followed by silver staining. **b,** A list of lipin-1-associated proteins identified by mass spectrometry (MS). Proteins were immunoprecipitated from Flag-tagged lipin-1-expressing *LPIN1*-KO MDA-MB-231 cells, followed by MS. **c, d,** Interaction between ectopically expressed lipin-1 and Src. Flag-lipin-1 was co-expressed with or without Src in HEK293T cells. Cells were lysed and subjected to IP against Flag (**c**) or HA (**d**), followed by immunoblotting. TCL, total cell lysate. **e,** GST pull-down assay was performed by using bacterially expressed His-lipin-1 with GST or GST-Src, followed by coomassie brilliant blue staining. **f,** SKI-606 and Dasatinib, Src inhibitors, impaired the association between Src and lipin-1. Flag-tagged lipin-1 was co-transfected with or without Src into HEK293T cells, which were then treated with or without SKI-606 or Dasatinib for 4 h. Flag-tagged lipin-1 was immunoprecipitated, followed by immunoblotting. **g,** Decreased association between endogenous Src and lipin-1 after treatment of Src inhibitors. MDA-MB-231 cells were stimulated with or without Src inhibitors (SKI-606 and Dasatinib) for 4 h. Lipin-1 was immunoprecipitated and subjected to immunoblotting. **h,** Kinase-active states of Src are required for its interaction with lipin-1. WT-Src, DN-Src (dominant-negative form K297R/Y529F), KD-Src (kinase dead form K297R) or Y529F-Src (constitutively activated form) was co-expressed with lipin-1 in HEK293T cells. Cells were lysed and subjected to IP against Flag, followed by immunoblotting. Source data are provided as a Source Data file.

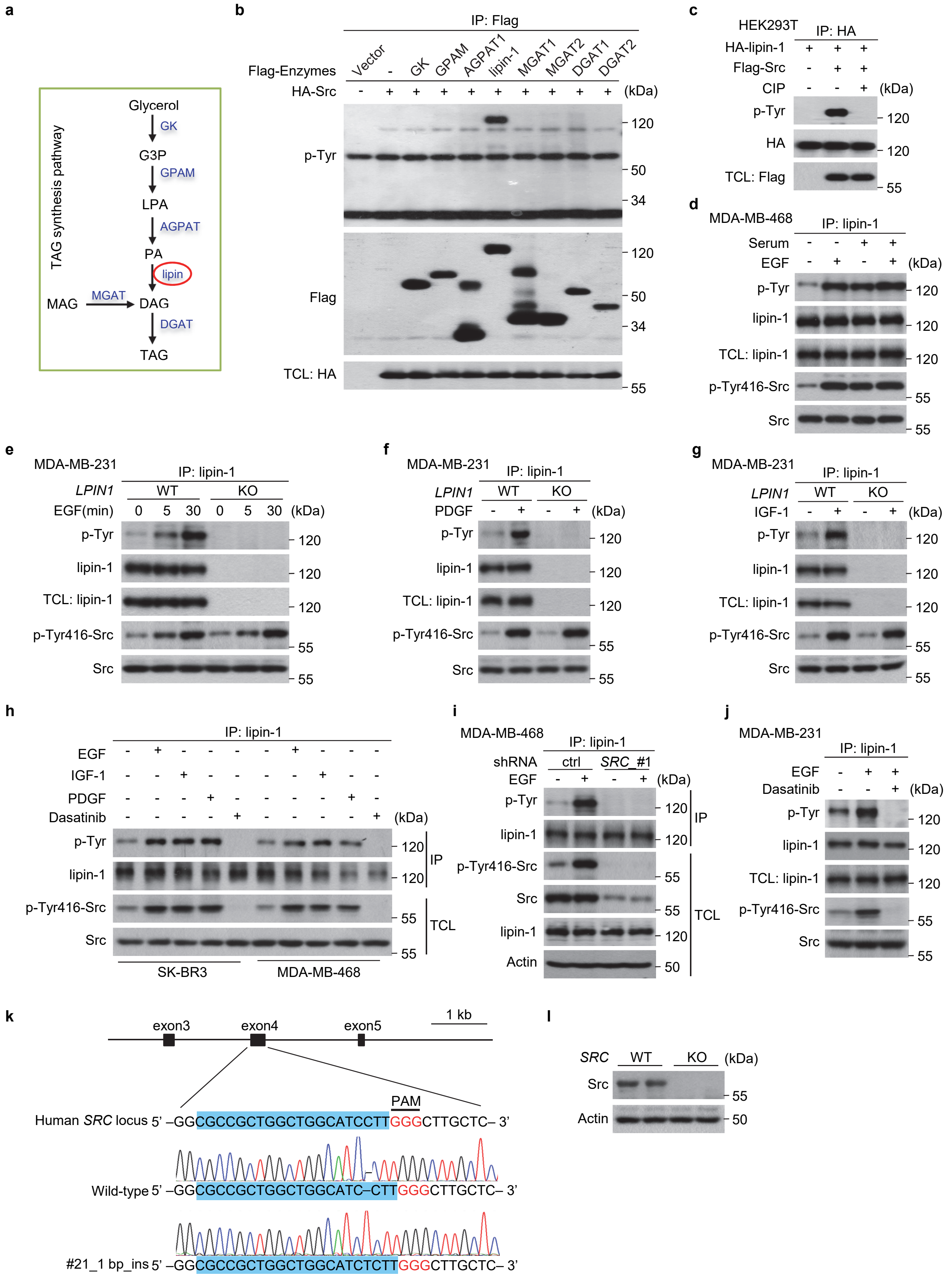
a
TAG synthesis pathway
Glycerol
GK
G3P
GPAM
LPA
AGPAT
PA
lipin
MAG
MGAT
DAG
DGAT
TAG
b
IP: Flag
Flag-Enzymes
Vector
-
GK
GPAM
AGPAT1
lipin-1
MGAT1
MGAT2
DGAT1
DGAT2
HA-Src
(kDa)
p-Tyr
Flag
TCL: HA
120
50
34
55
c
HEK293T
IP: HA
HA-lipin-1
Flag-Src
CIP
p-Tyr
HA
TCL: Flag
d
MDA-MB-468
IP: lipin-1
Serum
EGF
lipin-1
TCL: lipin-1
p-Tyr416-Src
Src
e
MDA-MB-231
LPIN1
WT
KO
EGF(min)
0
5
30
f
PDGF
g
IGF-1
h
Dasatinib
IP
TCL
SK-BR3
MDA-MB-468
i
shRNA
ctrl
SRC_#1
Actin
j
k
exon3
exon4
exon5
1 kb
PAM
Human SRC locus 5' –GGCGCCGCTGGCTGGCATCCTTGGGCTTGCTC– 3'
Wild-type 5' –GGCGCCGCTGGCTGGCATC–CTTGGGCTTGCTC– 3'
#21_1 bp_ins 5' –GGCGCCGCTGGCTGGCATCTCTTGGGCTTGCTC– 3'
l
SRC
WT
KO
Src
Actin
(kDa)
55
50

Supplementary Figure 3. lipin-1 is phosphorylated by Src.

a, Schematic diagram shows the enzymes involved in the triglyceride biosynthesis pathway. Lipin-1 is phosphorylated (circled in red). **b,** Src specifically phosphorylates lipin-1 but not other enzymes in the TAG synthesis pathway. Protein extracts of HEK293T after transfection were immunoprecipitated with anti-Flag antibody and immunoblotted as indicated. **c,** HEK293T cells were co-transfected with Flag-Src and HA-lipin-1, followed by immunoprecipitation for HA-lipin-1 and immunoblotting with a pan anti-phospho-tyrosine antibody (anti-p-Tyr) for detection of lipin-1 tyrosine phosphorylation. CIP, calf-intestinal alkaline phosphatase. **d,** Lipin-1 tyrosine phosphorylation stimulated by serum, or/and EGF. MDA-MB-468 cells were maintained in a serum-free medium for 4 h, and then treated with or without 100 ng/ml EGF, serum, or serum plus EGF for additional 30 min. **e,** Time-dependent tyrosine phosphorylation of lipin-1 after stimulation by EGF. WT and *LPIN1*-KO MDA-MB-231 cells were starved for 4 h, and then treated with EGF for 5 or 30 min. **f, g,** Lipin-1 tyrosine phosphorylation stimulated by platelet-derived growth factor (PDGF) or insulin-like growth factor-1 (IGF-1). MDA-MB-231 cells were maintained in a serum-free medium for 4 h, followed by stimulation with or without 20 ng/ml of PDGF (**f**) or 100 ng/ml of IGF-1 (**g**) for 30 min. **h,** Src dependent-lipin-1 phosphorylation stimulated by growth factor in breast cancer cells. SK-BR3 and MDA-MB-231 cells were maintained in a serum-free medium for 4 h, followed by stimulation with or without EGF, IGF-1 or PDGF for 30 min. **i,** Knockdown of *SRC* impairs tyrosine phosphorylation of lipin-1. MDA-MB-468 cells expressing shRNA against *SRC* or *Renilla* as a control were maintained in a serum-free medium for 4 h, followed by stimulation with or without EGF for 30 min. **j,** Src inhibitor blocks the phosphorylation of lipin-1 stimulated by EGF. MDA-MB-231 cells were maintained in a serum-free medium and pretreated with or without 1 μM Dasatinib for 4 h, followed by stimulation with or without EGF for 30 min. **k,** Generation of *SRC*-KO MDA-MB-231 cell line. Sequencing results showing the 1 bp insertion found in clone #21. **l,** Immunoblot analysis of total proteins of CRISPR control (WT) or S*RC*-KO (clone #21) MDA-MB-231 cells. (**d**-**j**) lipin-1 were immunoprecipitated, followed by immunoblotting as indicated. Source data are provided as a Source Data file.

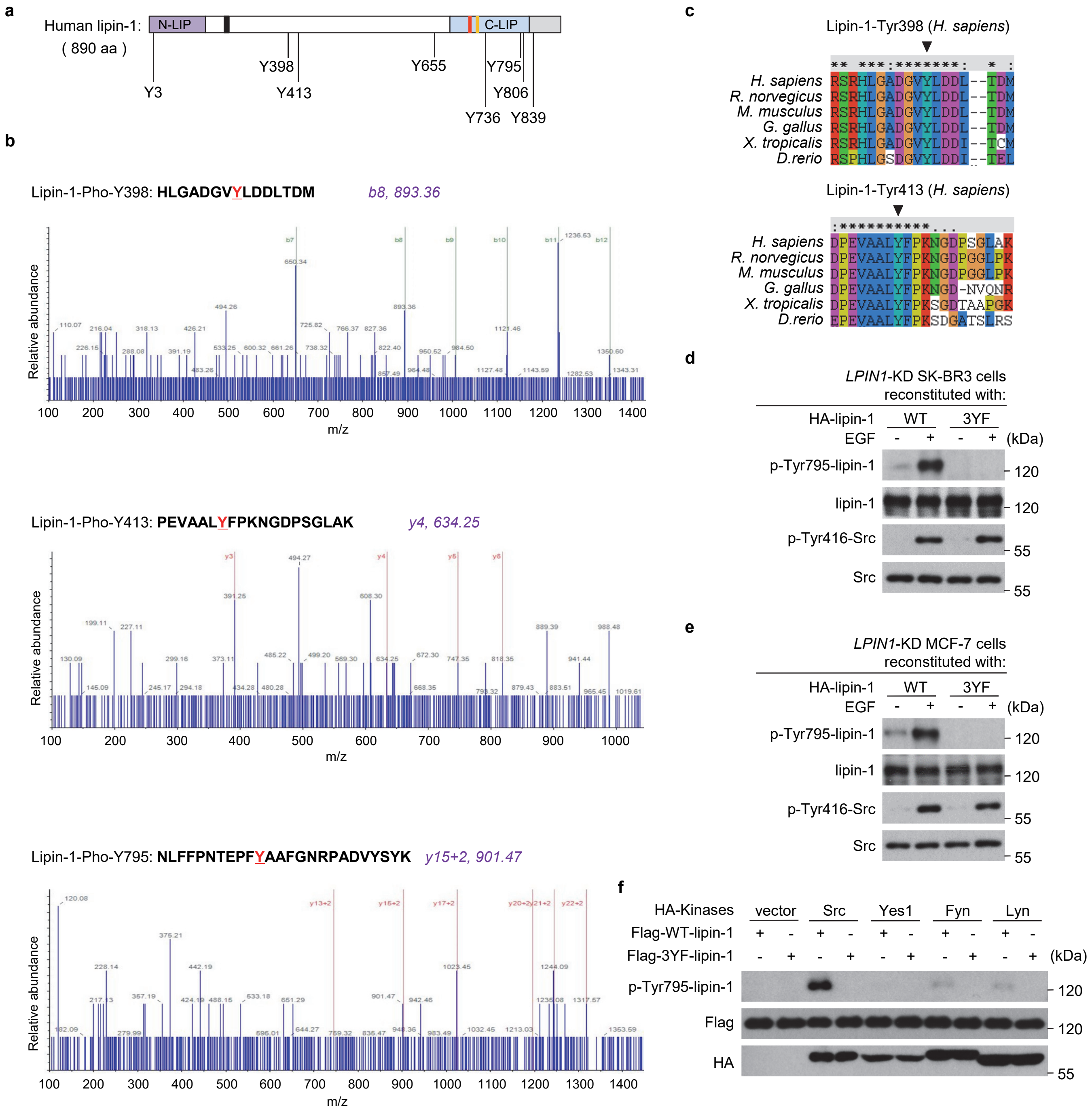

Supplementary Figure 4. Mass spectrometry analysis and sequence alignment of the tyrosine phosphorylation sites of lipin-1.

a, Mass spectrometric analysis for tyrosine phosphorylation sites on lipin-1. N-LIP, N-terminus lipin homology domain; C-NLP, C-terminal lipin-1 homology domain. **b,** Identification of Tyr398 (Y398), Tyr413 (Y413) and Tyr795 (Y795) phosphorylation on lipin-1 by liquid chromatography-mass spectrometry (LC-MS/MS) analysis. The representative mass spectrum of specific peptides containing phosphorylated residues (red). The mass/charge ratio (m/z) for determining each phosphorylated residue is also marked (purple). The labeled peaks show the masses of *y* or *b* ions of the phosphorylated peptides. **c,** Sequence alignment of the residues flanking Tyr398 and Tyr413 across different species. Arrow heads point to tyrosine residues corresponding to Tyr398 (top) and Tyr413 (bottom) in human lipin-1. **d, e,** 3YF-lipin-1 mutant fails to be phosphorylated in EGF-stimulated in breast cancer cells. *LPIN1*-KD SK-BR3 (**d**) or MCF-7 (**e**) cells stably expressing HA-tagged WT-lipin-1 or 3YF-lipin-1 were maintained in a serum-free medium for 4 h, followed with or without EGF treatment. Cell lysates were analyzed by immunoblotting. **f,** Fyn and Lyn show weaker ability in phosphorylating lipin-1 in HEK293T compared to Src. Source data are provided as a Source Data file.

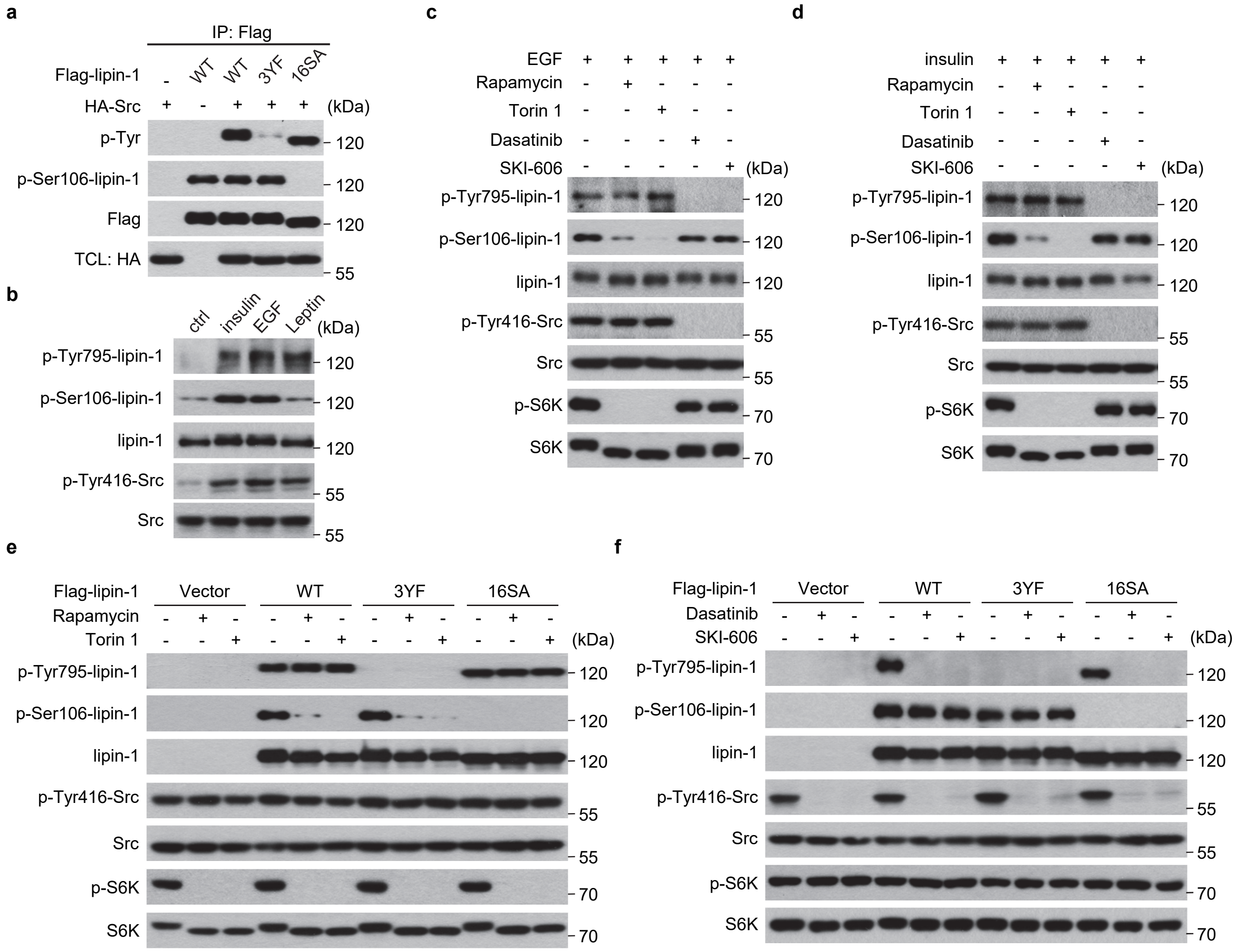

Supplementary Figure 5. Relationship between tyrosine phosphorylation and serine/threonine phosphorylation of lipin-1.

a, Dephosphorylation of lipin-1 does not affect its tyrosine phosphorylation by Src. Flag-tagged WT-lipin-1, 3YF-lipin-1 or 16SA-lipin-1 (serine/threonine phosphorylation-defective for mTOR) was co-expressed with or without Src in HEK293T cells, and then immunoprecipitated with anti-Flag antibody, followed by immunoblotting. **b,** EGF, insulin, and leptin effectively stimulate lipin-1 tyrosine phosphorylation. MDA-MB-231 cells were maintained in a serum-free medium for 4 h, and then treated with or without insulin, EGF or leptin for 30 min. **c, d,** Under EGF- and insulin-stimulated conditions, Src-mediated tyrosine phosphorylation of lipin-1 is not affected by the mTORC1 inhibitor, and Src inhibitor does not affect mTORC1-mediated serine phosphorylation. MDA-MB-231 cells were maintained in a serum-free medium and pretreated with or without mTOR inhibitor (rapamycin or Torin 1) or Src inhibitor (Dasatinib or SKI-606) for 4 h, followed by stimulation with EGF (**c**) or insulin (**d**) for 30 min. **e,** mTOR inhibitors do not affect Src-induced lipin-1 tyrosine phosphorylation. *LPIN1*-KO MDA-MB-231cells infected with vector, Flag-tagged WT-lipin-1, 3YF-lipin-1 or 16SA-lipin-1 were maintained in complete medium containing 10% FBS and treated with or without rapamycin or Torin 1. **f,** Src inhibitors do not affect mTORC1-mediated lipin-1 serine phosphorylation. *LPIN1*-KO MDA-MB-231 cells stably expressing vector, Flag-tagged WT-lipin-1, 3YF-lipin-1 or 16SA-lipin-1 were maintained in complete medium containing 10% FBS and treated with or without Dasatinib or SKI-606. Source data are provided as a Source Data file.

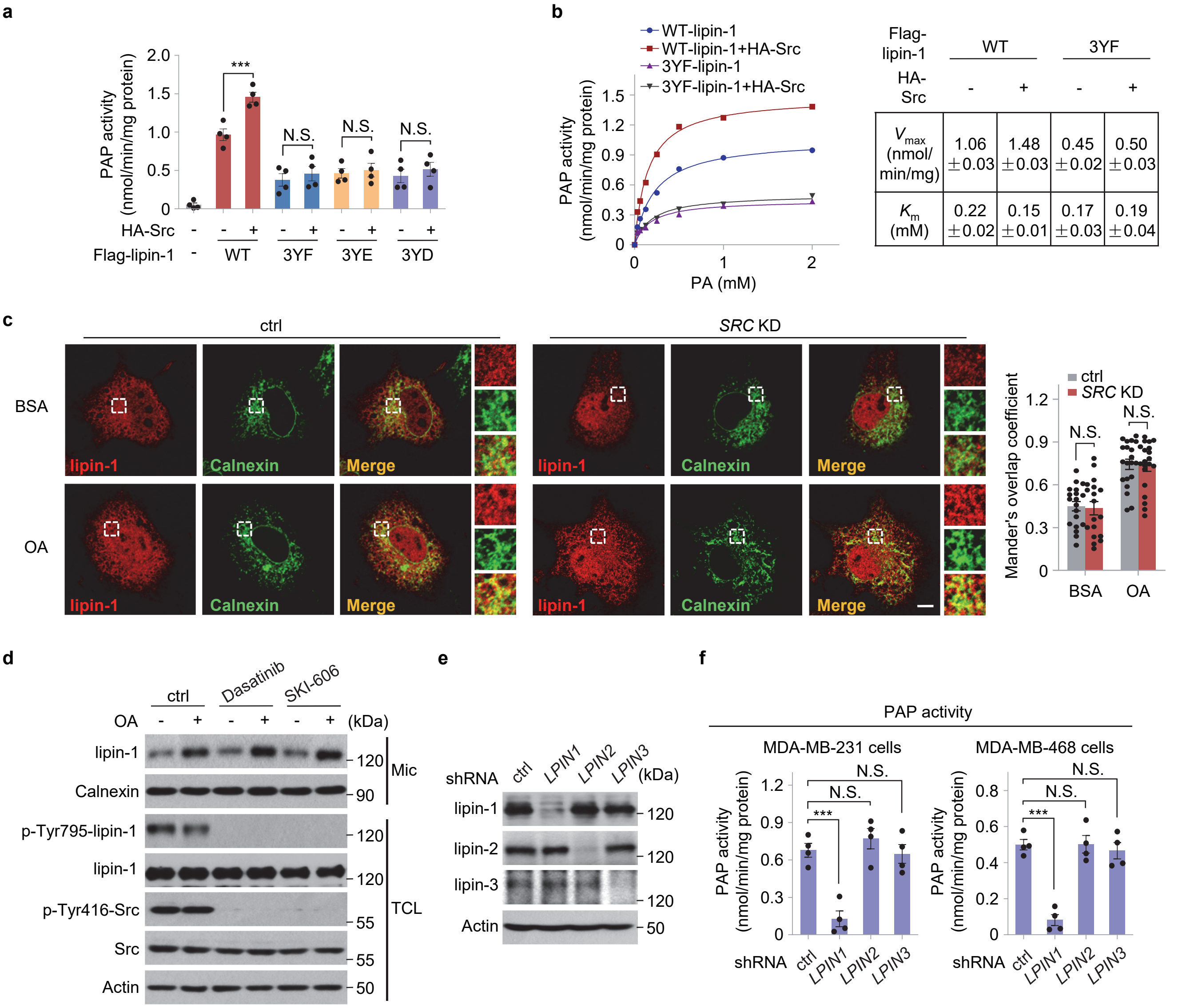

Supplementary Figure 6. Effects of Src-mediated phosphorylation on PAP activity and ER localization of lipin-1. **a,** Src-mediated phosphorylation enhances PAP activity of lipin-1. Flag tagged WT-lipin-1, 3YF-lipin-1, 3YE-lipin-1 or 3YD-lipin-1 was co-transfected with or without HA-Src into HEK293T. The enzymatic activities of immunoprecipitated Flag-lipin-1 were examined. $n = 4$ experiments. **b,** Enzymatic activities of WT or 3YF form of lipin-1 co-expressed with or without HA-tagged Src in HEK293T cells were assayed. Michaelis constants (*Km*) were determined from activities generated from at least 8 concentrations of substrates as indicated ($n = 4$ assays). **c,** Depletion of *SRC* does not affect oleic acid (OA)-induced lipin-1 translocation to endoplasmic reticulum (ER). MDA-MB-231 cells expressing shRNA against *SRC* (*SRC*-KD) or *Renilla* as a control were maintained in complete medium containing 10% FBS and treated with BSA or OA for 2 h. Calnexin is an ER marker. Mander's overlap coefficients between lipin-1 and Calnexin were graphed ($n = 19$ cells for ctrl treated with BSA group, n = 18 cells for *SRC*-KD treated with BSA group, n = 20 cells for ctrl or *SRC*-KD treated with OA group). Scale bars, 10 μm. **d,** Inhibition of Src does not affect OA induced lipin-1 translocation to ER membranes. MDA-MB-231 cells were maintained in complete medium containing 10% FBS and pretreated with DMSO or Src inhibitors (Dasatinib and SKI-606) for 4 h and co-treated with BSA or OA for 2 h and analyzed by fractionation, followed by immunoblotting. Calnexin, microsomal (Mic) marker. **e,** Lentivirus-mediated knockdown of *LPIN* in MDA-MB-231 cells. **f,** The PAP activity in MDA-MB-231 and MDA-MB-468 cells expressing shRNAs targeting *LPIN1* is diminished. $n = 4$ experiments. (**a-c, f**) were quantified in each independent experiment. Data are mean ± s.e.m.; ordinary one-way ANOVA, followed by Sidak in (**a**), or Dunnett in (**f**); ordinary two-way ANOVA, followed by Sidak in (**c**); $***P < 0.001$, N.S., not significant. Source data are provided as a Source Data file.

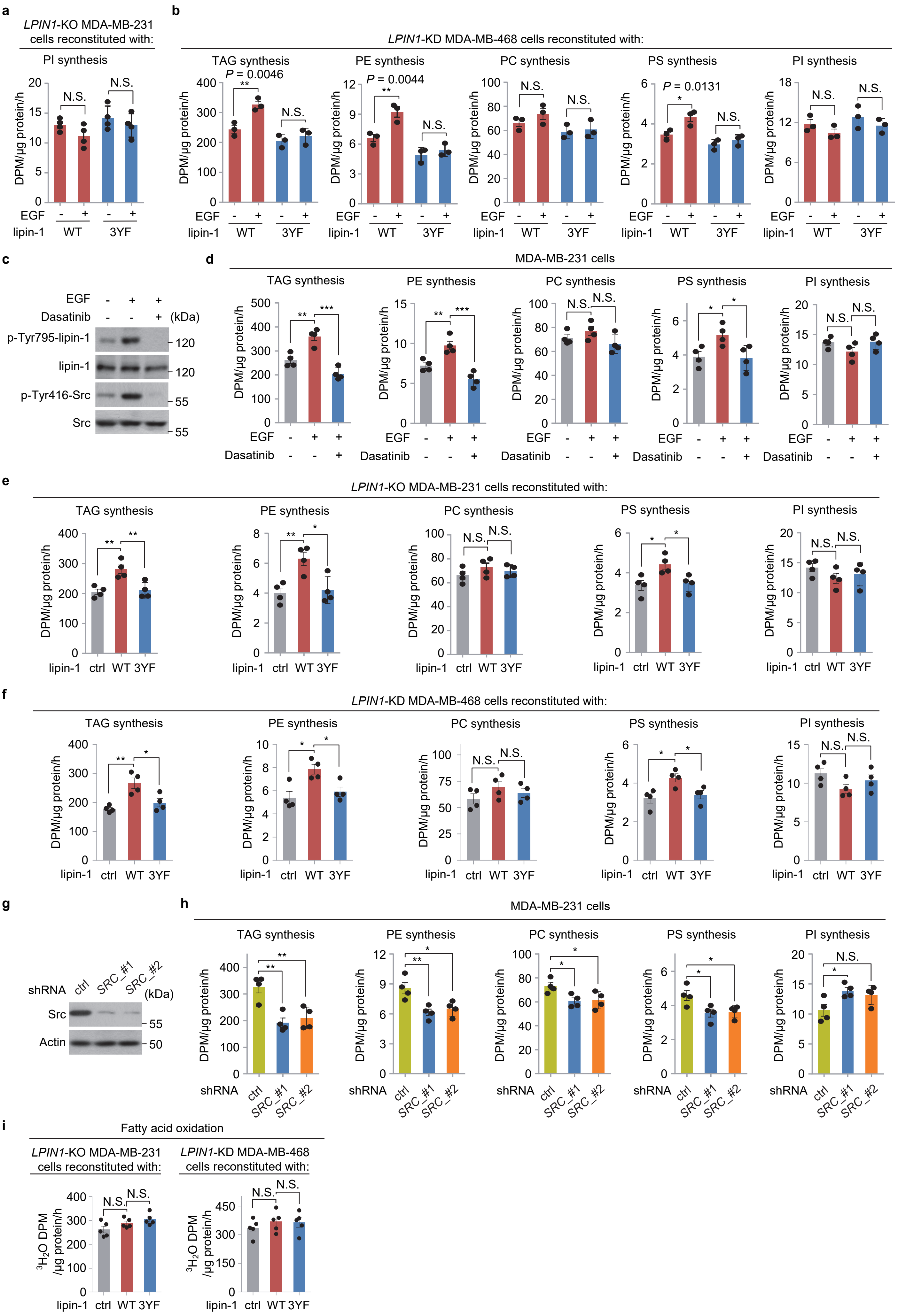
a
LPIN1-KO MDA-MB-231 cells reconstituted with:
PI synthesis
DPM/μg protein/h
EGF
lipin-1
WT
3YF
N.S.
b
LPIN1-KD MDA-MB-468 cells reconstituted with:
TAG synthesis
P = 0.0046
PE synthesis
P = 0.0044
PC synthesis
PS synthesis
P = 0.0131
PI synthesis
c
EGF
Dasatinib
(kDa)
p-Tyr795-lipin-1
lipin-1
p-Tyr416-Src
Src
120
120
55
55
d
MDA-MB-231 cells
TAG synthesis
PE synthesis
PC synthesis
PS synthesis
PI synthesis
e
LPIN1-KO MDA-MB-231 cells reconstituted with:
ctrl WT 3YF
f
LPIN1-KD MDA-MB-468 cells reconstituted with:
g
shRNA
ctrl
SRC_#1
SRC_#2
(kDa)
Src
Actin
55
50
h
MDA-MB-231 cells
TAG synthesis
PE synthesis
PC synthesis
PS synthesis
PI synthesis
i
Fatty acid oxidation
LPIN1-KO MDA-MB-231 cells reconstituted with:
LPIN1-KD MDA-MB-468 cells reconstituted with:
3H2O DPM /μg protein/h

Supplementary Figure 7. Src promotes lipin-1-mediated glycerolipid synthesis.

a, Phosphatidylinositol (PI) synthesis rates in *LPIN1*-KO MDA-MB-231 cells reconstituted with WT-lipin-1 or 3YF-lipin-1. Lipids from cells treated with ^{3}H-labeled OA and EGF were extracted and resolved by thin-layer chromatography (TLC), followed by quantification with scintillation counting. n = 4 experiments. **b,** TAG and phospholipid synthesis rates in *LPIN1*-KD MDA-MB-468 cells reconstituted with WT-lipin-1 or 3YF-lipin-1. TAG, triglyceride; PE, phosphatidylethanolamine; PC, phosphatidylcholine; PS, phosphatidylserine. n = 3 experiments. **c, d,** EGF-stimulated increase of glycerolipid synthesis is diminished by treating cells with Src inhibitor Dasatinib. Immunoblot analysis of protein (**c**) or glycerolipid synthesis rates (**d**) of MDA-MB-231 cells were maintained in a serum-free medium and pretreated with or without 1 μM Dasatinib for 4h, and then treated with or without EGF for 30 min. n = 4 experiments. **e, f,** Glycerolipid synthesis rates in *LPIN1*-KO MDA-MB-231(**e**) or *LPIN1*-KD MDA-MB-468 cells (**f**) reconstituted with vector (ctrl), WT-lipin-1 or 3YF-lipin-1 with ^{3}H-labeled OA treatment (n = 4 experiments). **g,** Lentivirus-mediated knockdown of *SRC* in MDA-MB-231 cells. **h,** TAG and phospholipid synthesis rates of MDA-MB-231 cells expressing shRNAs against *SRC* or *Renilla* as a control. Cells maintained in a normal medium were treated with ^{3}H-labeled OA (n = 4 experiments). **i,** Src-mediated phosphorylation of lipin-1 does not affect its fatty acid oxidation. Fatty acid oxidation rates in *LPIN1*-KO MDA-MB-231 and *LPIN1*-KD MDA-MB-468 cells infected with vector (ctrl), WT-lipin-1 or 3YF-lipin-1. Supernatant from cells treated with ^{3}H-labeled OA were transferred and mixed with 1.3 M perchloric acid, and subjected to centrifugateion, followed by quantification with scintillation counting (n = 5 experiments). (**a, b, d-f, h, i**) were quantified in each independent experiment. Data are mean ± s.e.m.; ordinary two-way ANOVA followed by Sidak in (**a, b**); ordinary one-way ANOVA followed by Tukey in (**d-f, i**), or by Dunnett in (**h**); $^*P < 0.05$, $^{**}P < 0.01$, $^{***}P < 0.001$, N.S., not significant. Source data are provided as a Source Data file.

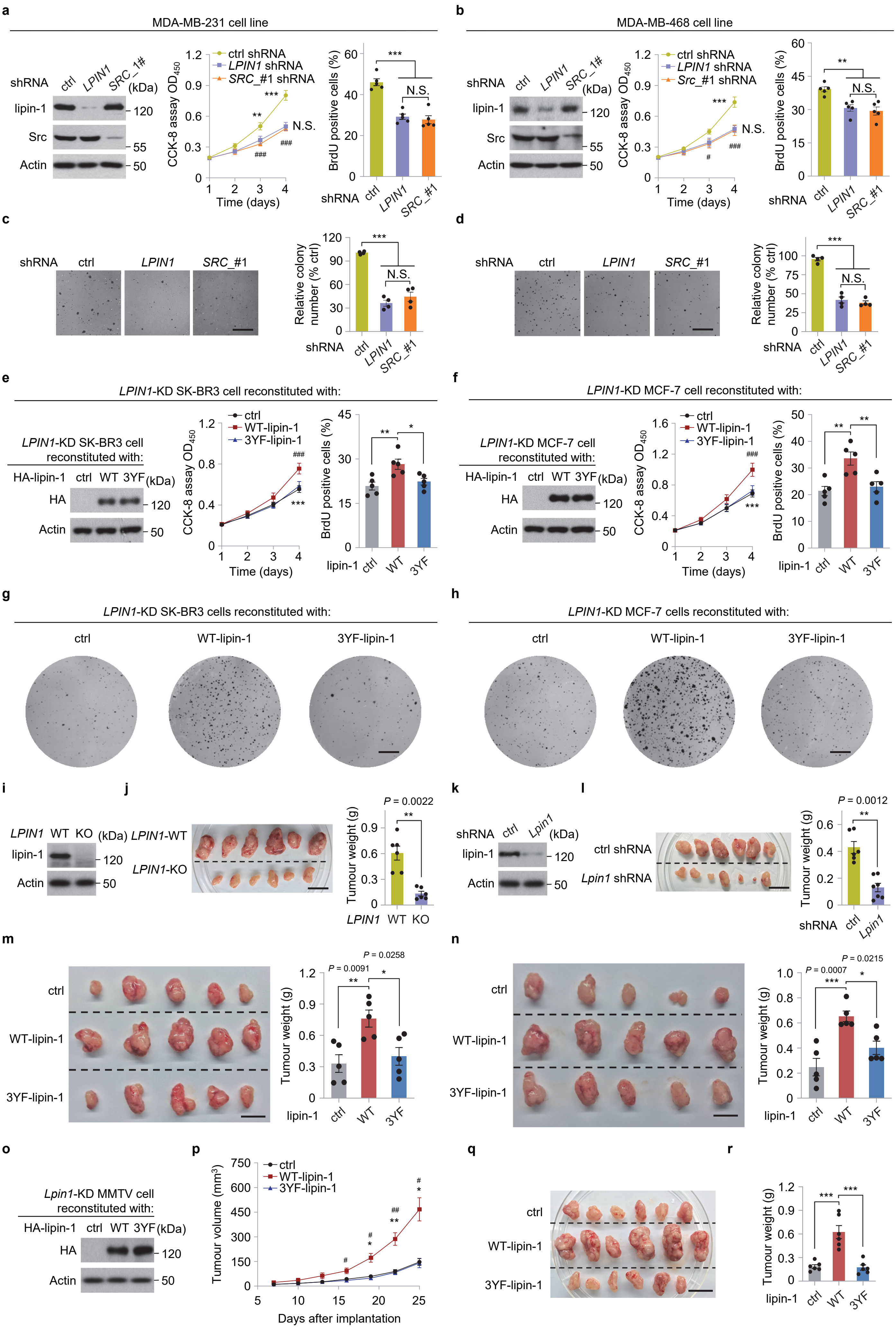

a
MDA-MB-231 cell line
shRNA ctrl LPIN1 SRC_1# (kDa)
lipin-1 120
Src 55
Actin 50
CCK-8 assay OD450
ctrl shRNA
LPIN1 shRNA
SRC_#1 shRNA
Time (days)
N.S.
BrdU positive cells (%)
shRNA ctrl LPIN1 SRC_#1
b
MDA-MB-468 cell line
shRNA ctrl LPIN1 SRC_1# (kDa)
lipin-1 120
Src 55
Actin 50
CCK-8 assay OD450
ctrl shRNA
LPIN1 shRNA
Src_#1 shRNA
Time (days)
N.S.
BrdU positive cells (%)
shRNA ctrl LPIN1 SRC_#1
c
shRNA ctrl LPIN1 SRC_#1
Relative colony number (% ctrl)
N.S.
shRNA ctrl LPIN1 SRC_#1
d
shRNA ctrl LPIN1 SRC_#1
Relative colony number (% ctrl)
N.S.
shRNA ctrl LPIN1 SRC_#1
e
LPIN1-KD SK-BR3 cell reconstituted with:
LPIN1-KD SK-BR3 cell reconstituted with:
HA-lipin-1 ctrl WT 3YF (kDa)
HA 120
Actin 50
CCK-8 assay OD450
ctrl
WT-lipin-1
3YF-lipin-1
Time (days)
BrdU positive cells (%)
lipin-1 ctrl WT 3YF
f
LPIN1-KD MCF-7 cell reconstituted with:
LPIN1-KD MCF-7 cell reconstituted with:
HA-lipin-1 ctrl WT 3YF (kDa)
HA 120
Actin 50
CCK-8 assay OD450
ctrl
WT-lipin-1
3YF-lipin-1
Time (days)
BrdU positive cells (%)
lipin-1 ctrl WT 3YF
g
LPIN1-KD SK-BR3 cells reconstituted with:
ctrl WT-lipin-1 3YF-lipin-1
h
LPIN1-KD MCF-7 cells reconstituted with:
ctrl WT-lipin-1 3YF-lipin-1
i
LPIN1 WT KO (kDa)
lipin-1 120
Actin 50
j
LPIN1-WT
LPIN1-KO
P = 0.0022
Tumour weight (g)
LPIN1 WT KO
k
shRNA ctrl Lpin1 (kDa)
lipin-1 120
Actin 50
l
ctrl shRNA
Lpin1 shRNA
P = 0.0012
Tumour weight (g)
shRNA ctrl Lpin1
m
ctrl
WT-lipin-1
3YF-lipin-1
P = 0.0258
P = 0.0091
Tumour weight (g)
lipin-1 ctrl WT 3YF
n
ctrl
WT-lipin-1
3YF-lipin-1
P = 0.0215
P = 0.0007
Tumour weight (g)
lipin-1 ctrl WT 3YF
o
Lpin1-KD MMTV cell reconstituted with:
HA-lipin-1 ctrl WT 3YF (kDa)
HA 120
Actin 50
p
Tumour volume (mm3)
ctrl
WT-lipin-1
3YF-lipin-1
Days after implantation
q
ctrl
WT-lipin-1
3YF-lipin-1
r
Tumour weight (g)
lipin-1 ctrl WT 3YF

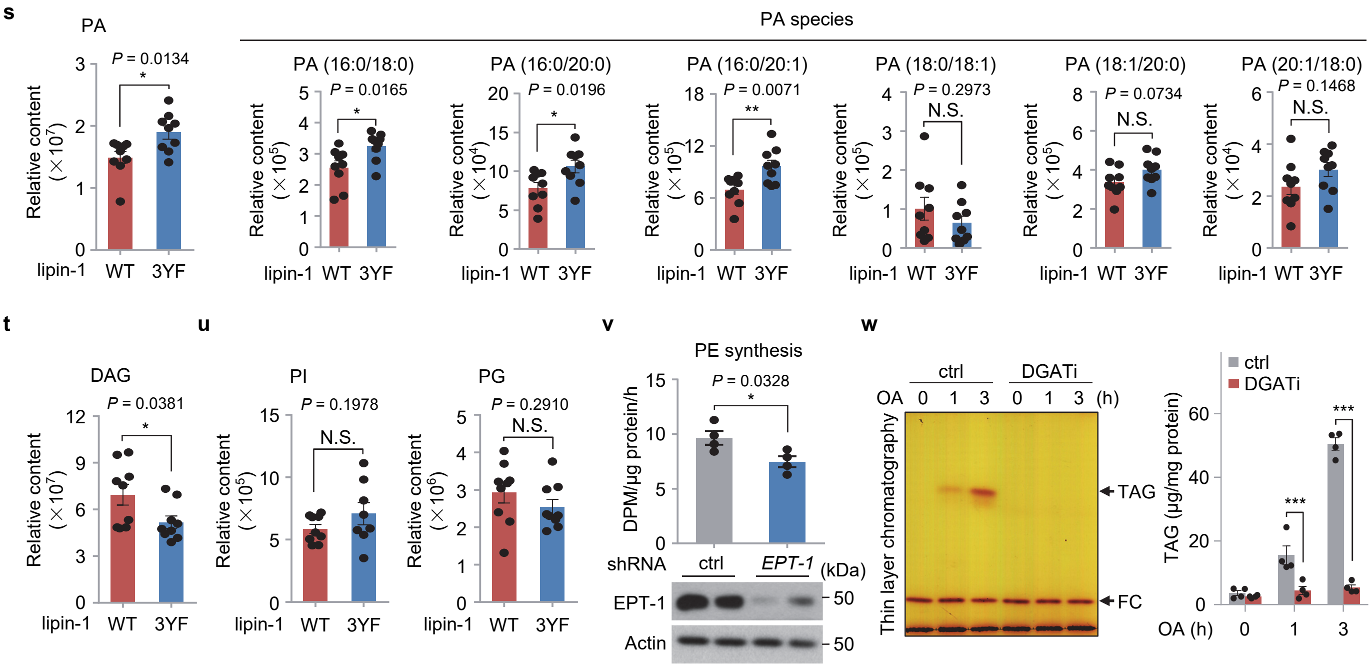
s
PA
P = 0.0134
*
Relative content (×10^7)
lipin-1 WT 3YF
PA species
PA (16:0/18:0)
P = 0.0165
*
Relative content (×10^5)
PA (16:0/20:0)
P = 0.0196
*
Relative content (×10^4)
PA (16:0/20:1)
P = 0.0071
**
Relative content (×10^5)
PA (18:0/18:1)
P = 0.2973
N.S.
Relative content (×10^5)
PA (18:1/20:0)
P = 0.0734
N.S.
Relative content (×10^5)
PA (20:1/18:0)
P = 0.1468
N.S.
Relative content (×10^4)
t
DAG
P = 0.0381
*
Relative content (×10^7)
lipin-1 WT 3YF
u
PI
P = 0.1978
N.S.
Relative content (×10^5)
PG
P = 0.2910
N.S.
Relative content (×10^6)
v
PE synthesis
P = 0.0328
*
DPM/μg protein/h
shRNA ctrl EPT-1
(kDa)
EPT-1
Actin
50
w
ctrl DGATi
OA 0 1 3 0 1 3 (h)
Thin layer chromatography
TAG
FC
ctrl
DGATi
***
TAG (μg/mg protein)
OA (h) 0 1 3

Supplementary Figure 8. Tyrosine phosphorylation of lipin-1 by Src facilitates cell proliferation, anchorage-independent growth and tumorigenesis.

a, b, Knockdown of *LPIN1* and *SRC* similarly suppresses cell proliferation. The CCK-8 assay and BrdU incorporation assay were performed to determine viable cell numbers in MDA-MB-231 (**a**) and MDA-MB-468 (**b**) cells. (Middle graph of **a**) ctrl shRNA versus *LPIN1* shRNA, $^{**}P = 0.0042$ (day 3), $^{***}P < 0.001$ (day 4); ctrl shRNA versus *SRC*_1# shRNA, $^{\#\#\#}P < 0.001$ (day 3 and day 4); *LPIN1* shRNA versus *SRC*_1# shRNA, N.S., not significant. (Middle graph of **b**) ctrl shRNA versus *LPIN1* shRNA, $^{***}P < 0.001$ (day 4); ctrl shRNA versus *SRC*_1# shRNA, $^{\#}P = 0.0408$ (day 3), $^{\#\#\#}P < 0.001$ (day 4); *LPIN1* shRNA versus *SRC*_1# shRNA, N.S., not significant. **c, d,** Soft agar colony formation was performed with MDA-MB-231 cells or MDA-MB-468 cells expressing shRNAs targeting *LPIN1* or *SRC* or *Renilla* as a control, and quantification of relative colony number is shown on the right. The cells were maintained in complete medium containing 10% FBS. The scale bars represent 5 mm. **e, f,** Re-expression of WT-lipin-1 but not 3YF-lipin-1 restored the proliferation of breast cancer cells. SK-BR3 (**e**) or MCF-7 (**f**) cells knocked-down of *LPIN1* (*LPIN1*-KD) infected with empty vector (ctrl), WT-lipin-1 or 3YF-lipin-1 and were maintained in complete medium containing 10% FBS. Western blotting of proteins of *LPIN1*-KD cells infected with ctrl, WT-lipin-1 or 3YF-lipin-1 lentiviruses (left graph of **e** and **f**,). The CCK-8 assay ($n = 4$ experiments; middle graphs) and BrdU incorporation assay ($n = 5$ experiments; right graphs) were performed to determine viable cell number. (middle graph of **e**) ctrl versus WT-lipin-1, $^{***}P < 0.001$ (day 4); WT-lipin-1 versus 3YF-lipin-1, $^{\#\#\#}P < 0.001$ (day 4). (middle graph of **f**) ctrl versus WT-lipin-1, $^{***}P < 0.001$ (day 4); WT-lipin-1 versus 3YF-lipin-1, $^{\#\#\#}P < 0.001$ (day 4). **g, h,** The soft agar colony formation were performed with *LPIN1*-KD SK-BR3 (**g**) or MCF-7 (**h**) cells reconstituted with WT-lipin-1, 3YF-lipin-1 or empty vector as a control. The cells were maintained in complete medium containing 10% FBS. Scale bars, 5 mm. **i,** Immunoblot analysis of proteins of WT or *LPIN1*-KO MDA-MB-231 cells. **j,** Representative images and tumour weights of mouse xenograft tumours from nude mice implanted with *LPIN1*-KO or WT MDA-MB-231 cells. $n = 6$ mice per group. Scale bar, 10 mm. **k,** Knockdown of *Lpin1* in MMTV-PyVT mammary carcinoma-derived murine mammary tumour (MMTV) cells. MMTV tumour cells established from a mammary tumour of MMTV-PyVT mouse. **l,** Representative photographs and tumour weights of mouse orthotopic xenograft tumour from FVB/N female mice implanted with MMTV tumour cells expressing shRNA targeting *Lpin1* (*Lpin1* shRNA, $n = 7$) or *Renilla* as a control (ctrl shRNA, $n = 6$). Scale bar, 10 mm. **m, n,** Representative images and tumor weights of mouse xenograft tumors from mouse xenograft implanted with *LPIN1*-KD SK-BR3 (**m**) or MCF-7 (**n**) cells expressing vector (ctrl), WT-lipin-1 or 3YF-lipin-1 in nude mice ($n = 5$ mice per group). **o,** Immunoblotting analysis of proteins of *Lpin1*-KD MMTV cells infected with vector (ctrl), WT-lipin-1 or 3YF-lipin-1. **p,** Orthotopic xenograft tumor growth in mice. Volumes of tumors generated by inoculating *Lpin1*-KD MMTV tumor cells expressing ctrl, WT-lipin-1 and 3YF-lipin-1 into mammary fat pad of FVB/N mice were measured on different days. $n = 6$ mice per group. Ctrl versus WT-lipin-1, $^{*}P = 0.0169$ (day 19), $^{**}P = 0.0061$ (day 22), $^{*}P = 0.012$ (day 25); WT-lipin-1 versus 3YF-lipin-1, $^{\#}P = 0.0441$ (day 16), $^{\#}P = 0.0115$ (day 19), $^{\#\#}P = 0.0049$ (day 22), $^{\#}P = 0.0101$ (day 25). **q, r,** Representative photographs and tumor weights of mouse orthotopic xenograft tumors from (**p**). Scale bar, 10 mm. **s,** Relative levels of phosphatidic acid (PA) (left) and individual PA species (right) of xenograft tumors from nude mice implanted with *LPIN1*-KO MDA-MB-231 cells reconstituted with WT-lipin-1 or 3YF-lipin-1. $n = 9$ mice per group. **t, u,** Relative levels of diacylglycerol (DAG) or phospholipid of mouse xenograft tumors from nude mice implanted with *LPIN1*-KO MDA-MB-231 cells reconstituted with WT-lipin-1 or 3YF-lipin-1. PI, phosphatidylinositol; PG, phosphatidylglycerol. $n = 9$ individuals for each group. The whole list of lipids identified and statistical analysis methods can be found in **Supplementary Data 1**. **v,** Knockdown of *EPT-1* and PE synthesis rates in MDA-MB-231 cells. Lipids from cells treated with ^{3}H-labeled OA and EGF were extracted and resolved by thin-layer chromatography (TLC), followed by quantification with scintillation counting. **w,** TAG levels of MDA-MB-231 cells treated with DMSO (ctrl) or DGAT inhibitors (PF-04620110 and PF-06424439) with OA stimulation ($n = 4$ experiments). (**a-f, v, w**) were quantified in each independent experiment. (**j, l, m, n, q**) were quantified for each xenograft tumour. Data are mean ± s.e.m.; two-way ANOVA (repeated measure) followed by Tukey in (middle graph of **a, b, e, f,** and **p**); ordinary two-way ANOVA followed by Tukey in (**w**); ordinary one-way ANOVA followed by Tukey in (right graph of **a-f, m, n** and **r**); two-tailed Mann-Whitney test in (**j, l**); two-tailed unpaired Student's *t*-test in (**s-v**). $^{*}P < 0.05$, $^{**}P < 0.01$, $^{***}P < 0.001$, N.S., not significant. Source data are provided as a Source Data file.

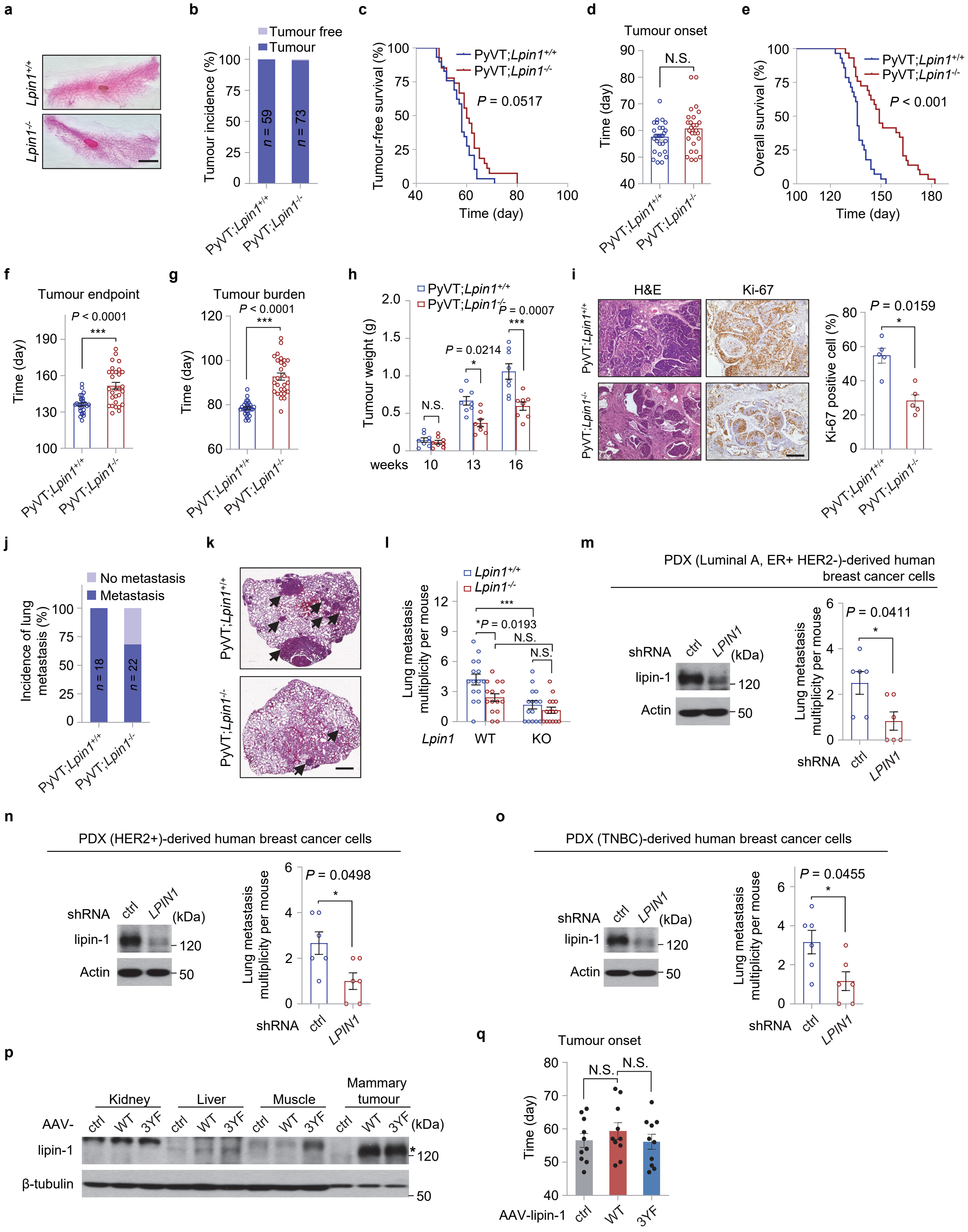
a
Lpin1+/+
Lpin1-/-
b
Tumour free
Tumour
Tumour incidence (%)
n = 59
n = 73
PyVT;Lpin1+/+
PyVT;Lpin1-/-
c
Tumour-free survival (%)
PyVT;Lpin1+/+
PyVT;Lpin1-/-
P = 0.0517
Time (day)
d
Tumour onset
N.S.
Time (day)
e
Overall survival (%)
P < 0.001
Time (day)
f
Tumour endpoint
P < 0.0001
***
Time (day)
g
Tumour burden
P < 0.0001
***
Time (day)
h
Tumour weight (g)
P = 0.0007
P = 0.0214
N.S.
weeks
i
H&E
Ki-67
Ki-67 positive cell (%)
P = 0.0159
j
No metastasis
Metastasis
Incidence of lung metastasis (%)
n = 18
n = 22
k
l
Lpin1+/+
Lpin1-/-
Lung metastasis multiplicity per mouse
*P = 0.0193
N.S.
Lpin1
WT
KO
m
PDX (Luminal A, ER+ HER2-)-derived human breast cancer cells
shRNA
ctrl
LPIN1
(kDa)
lipin-1
Actin
P = 0.0411
n
PDX (HER2+)-derived human breast cancer cells
P = 0.0498
o
PDX (TNBC)-derived human breast cancer cells
P = 0.0455
p
Kidney
Liver
Muscle
Mammary tumour
AAV-
ctrl
WT
3YF
lipin-1
β-tubulin
q
Tumour onset
Time (day)
AAV-lipin-1

Supplementary Figure 9. Src-mediated lipin-1 tyrosine phosphorylation facilitates tumour progression.
a, Representative images of the mammary glands from 2-month-old *Lpin1*$^{+/+}$ and *Lpin1*$^{-/-}$ female mice, visualized by carmine alum staining. Scale bars, 5 mm. **b,** Tumour incidences in PyVT;*Lpin1*$^{+/+}$ (*n* = 59) and PyVT;*Lpin1*$^{-/-}$ (*n* = 73) mice. Bars are the percentages of tumour incidences. **c,** Tumour-free survival of the PyVT;*Lpin1*$^{+/+}$ (*n* = 29) and PyVT;*Lpin1*$^{-/-}$ (*n* = 27) mice. The difference between the two genotypes is non-significant. **d,** The day of the appearance of palpable tumour. *n* = 27-29 mice per group. **e,** Kaplan-Meier survival analysis of the PyVT;*Lpin1*$^{+/+}$ (*n* = 28 mice, median overall survival 136 days) and the PyVT;*Lpin1*$^{-/-}$ (*n* = 29 mice, median overall survival 149 days). **f, g,** The day of the tumour endpoint (**f**) or tumour burden (**g**). *n* = 27-28 mice per group. **h,** Tumour weights of PyVT;*Lpin1*$^{+/+}$ and PyVT;*Lpin1*$^{-/-}$ mice were measured at week 10, 13 and 16. *n* = 8 mice per group. **i,** Representative hematoxylin and eosin (H&E) and Ki-67 staining of mammary sections isolated from PyVT;*Lpin1*$^{+/+}$ and PyVT;*Lpin1*$^{-/-}$ mice (*n* = 5 per group), and quantification of Ki-67-positive cells per view field is shown on the right. Scale bars, 100 μm. **j,** Incidence of lung metastasis. Bars are the percentages of mice with lung metastasis. *n* = 18 mice for PyVT;*Lpin1*$^{+/+}$ group and *n* = 22 for PyVT;*Lpin1*$^{-/-}$ group. **k,** Representative H&E stained lung sections. Arrowheads (black) indicate clusters of metastatic cells in the lung. Scale bars, 20 μm. **l,** Depletion of *Lpin1* diminishes the differences of lung metastases between WT and *Lpin1*$^{-/-}$ mice. 1×10^5 WT or *Lpin1*-KO MMTV cells were injected intravenously into either *Lpin1*$^{+/+}$ or *Lpin1*$^{-/-}$ mice. *n* = 15 mice per group. **m-o,** Knockdown of *LPIN1* suppresses lung metastasis. The patient-derived xenograft (PDX)-derived Lunimal A (ER+, HER2-) or triple negative breast cancer (TNBC) or HER2 positive breast cancer (HER2+) cells expressing shRNA targeting *LPIN1* (*LPIN1* shRNA) or *Renilla* as a control (ctrl shRNA) were injected through tail vein of six-week-old immunocompromised NOD-SCID female mice. At 8 weeks post-injection, lung tissues were fixed with 4% paraformaldehyde and paraffin embedded for histology study. Metastasis multiplicity were determined by H&E staining. **p,** Immunoblot analysis of the extracts of different tissues isolated from PyVT;*Lpin1*$^{-/-}$ female mice infected with AAV-vector (ctrl), AAV-WT-lipin-1 or AAV-3YF-lipin-1. Asterisk indicates lipin-1 protein. **q,** The days of the appearance of palpable tumour. PyVT;*Lpin1*$^{-/-}$ female mice infected with AAV-vector (ctrl), AAV-WT-lipin-1 or AAV-3YF-lipin-1. *n* = 10 mice per group. (**c, e**) analyzed using log rank (Mantel-Cox) test. Data are mean ± s.e.m.; two-tailed Mann-Whitney test in (**d, f, g, i, m-o**); ordinary two-way ANOVA, followed by Sidak in (**h**), or Tukey in (**l**); one-way ANOVA (repeated measure), followed by Tukey in (**q**). $^{***}P < 0.001$, N.S., not significant. Source data are provided as a Source Data file.

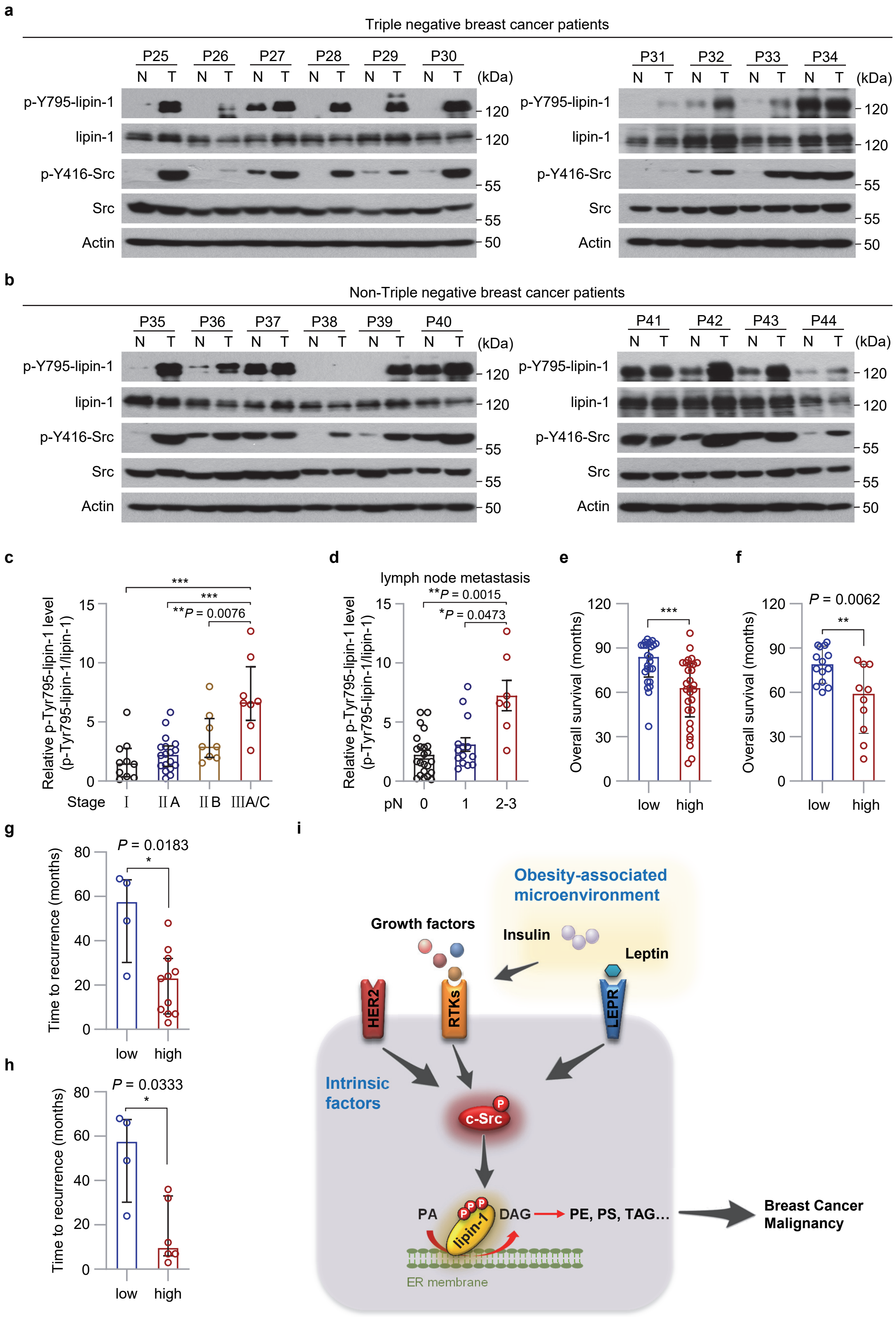

a
Triple negative breast cancer patients
P25 P26 P27 P28 P29 P30
N T N T N T N T N T N T (kDa)
P31 P32 P33 P34
N T N T N T N T (kDa)
p-Y795-lipin-1
lipin-1
p-Y416-Src
Src
Actin
120
120
55
55
50
b
Non-Triple negative breast cancer patients
P35 P36 P37 P38 P39 P40
N T N T N T N T N T N T (kDa)
P41 P42 P43 P44
N T N T N T N T (kDa)
c
Relative p-Tyr795-lipin-1 level (p-Tyr795-lipin-1/lipin-1)
***
***
**P = 0.0076
Stage I IIA IIB IIIA/C
d
lymph node metastasis
**P = 0.0015
*P = 0.0473
pN 0 1 2-3
e
Overall survival (months)
***
low high
f
P = 0.0062
**
g
Time to recurrence (months)
P = 0.0183
*
h
P = 0.0333
i
Obesity-associated microenvironment
Growth factors
Insulin
Leptin
HER2
RTKs
LEPR
Intrinsic factors
c-Src
P
PA
lipin-1
DAG
PE, PS, TAG…
ER membrane
Breast Cancer Malignancy

Supplementary Figure 10. Src-mediated lipin-1 tyrosine phosphorylation promotes human breast cancer progression. **a, b,** Immunoblot analysis of p-Tyr795-lipin-1 and p-Tyr416-Src in breast tumour specimens and matched adjacent normal tissues in triple negative breast cancer (TNBC, **a**) or non-TNBC (**b**) patients. N, tumour-adjacent normal tissue; T, tumour; P25, patient 25. **c, d,** Analysis of p-Tyr795-lipin-1 in different clinical stages (**c**) or lymph node metastasis (**d**) of the breast cancer patients. **e, f,** Decreased overall survival in p-Tyr795-lipin-1-high patients. Data of p-Tyr795-lipin-1 levels were grouped by median (**e**) or quartile (**f**). **g, h,** Decreased time to recurrence in p-Tyr795-lipin-1-high patients. P-Tyr795-lipin-1 levels were grouped by median (**g**) or quartile (**h**). **i,** Model of Src-regulated lipin-1 phosphorylation in response to different stimuli and breast cancer progression. Several elements of this image were adopted and modified from Servier Medical Art by Servier (https://smart.servier.com/) licensed under a Creative Commons Attribution 3.0 Unported License (https://creativecommons.org/licenses/by/3.0/). (**c-h**) Data are median with interquartile range, two-tailed Mann-Whitney test in (**e-h**), ordinary one-way ANOVA followed by Tukey in (**c, d**). ***P < 0.001. Source data are provided as a Source Data file.
