# Supplementary material for: Proto-oncogene Src links lipogenesis via lipin-1 to breast cancer malignancy
Source: Nat Commun. 2020 Nov 17;11:5842. doi: 10.1038/s41467-020-19694-w (PMC7672079; doi:10.1038/s41467-020-19694-w)
Supplement: Supplementary file 2 — Description of Additional Supplementary Files [file 41467_2020_19694_MOESM2_ESM.pdf]

Description of Additional Supplementary Files

File Name: Supplementary Data 1
Description: Lipidomic profiling of xenograft tumours from nude mice implanted with LPIN1-KO MDA-MB-231 cells reconstituted with WT-lipin-1 or 3YF-lipin-1 by LC-MS/MS.

File Name: Supplementary Data 2
Description: Characteristics of breast cancer patients whose tumours were used for PDX construction.

File Name: Supplementary Data 3
Description: Characteristics of breast cancer tissues from 44 patients (from Sun Yat-Sen University Cancer Center).

File Name: Supplementary Data 4
Description: Overall survival and relapse-free survival of patients for breast cancer tissue array (from Xijing Hospital).

File Name: Supplementary Data 5
Description: A list of primers used in our study.
