# Supplementary material for: Proto-oncogene Src links lipogenesis via lipin-1 to breast cancer malignancy
Source: Nat Commun. 2020 Nov 17;11:5842. doi: 10.1038/s41467-020-19694-w (PMC7672079; doi:10.1038/s41467-020-19694-w)
Supplement: Supplementary file 8 — Reporting Summary [file 41467_2020_19694_MOESM8_ESM.pdf]

nature research

Corresponding author(s): Sheng-Cai Lin

Last updated by author(s): Sep 4, 2020

Reporting Summary

Nature Research wishes to improve the reproducibility of the work that we publish. This form provides structure for consistency and transparency in reporting. For further information on Nature Research policies, see our Editorial Policies and the Editorial Policy Checklist.

Statistics

For all statistical analyses, confirm that the following items are present in the figure legend, table legend, main text, or Methods section.

n/a	Confirmed	
☐	☒	The exact sample size (*n*) for each experimental group/condition, given as a discrete number and unit of measurement
☐	☒	A statement on whether measurements were taken from distinct samples or whether the same sample was measured repeatedly
☐	☒	The statistical test(s) used AND whether they are one- or two-sided<br>*Only common tests should be described solely by name; describe more complex techniques in the Methods section.*
☒	☐	A description of all covariates tested
☐	☒	A description of any assumptions or corrections, such as tests of normality and adjustment for multiple comparisons
☐	☒	A full description of the statistical parameters including central tendency (e.g. means) or other basic estimates (e.g. regression coefficient) AND variation (e.g. standard deviation) or associated estimates of uncertainty (e.g. confidence intervals)
☐	☒	For null hypothesis testing, the test statistic (e.g. *F*, *t*, *r*) with confidence intervals, effect sizes, degrees of freedom and *P* value noted<br>*Give P values as exact values whenever suitable.*
☒	☐	For Bayesian analysis, information on the choice of priors and Markov chain Monte Carlo settings
☒	☐	For hierarchical and complex designs, identification of the appropriate level for tests and full reporting of outcomes
☐	☒	Estimates of effect sizes (e.g. Cohen's *d*, Pearson's *r*), indicating how they were calculated

Our web collection on statistics for biologists contains articles on many of the points above.

Software and code

Policy information about availability of computer code

Data collection	Image J 1.44p and GraphPad Prism 8.0.2 were used for data collection.
Data analysis	Data were analysed using GraphPad Prism 8.0.2 built-in tests. For experiments with only two groups, two-tailed Student's t test or Mann-Whitney test was used for statistical comparisons based on the results of normality test of each group of data. Welch's correction was used for unequal variances. One-way or two-way ANOVA (ordinary or repeated measure) with post-tests (Tukey, Sidak, Dunnett) as indicated in the figure legends was used for comparison of data with multiple groups. Geisser-Greenhouse's correction was used for ANOVA tests where applicable. Pearson's correlation test was used for measure of the strength of the association between two variables. Log rank (Mantel-Cox) test was used to compare the survival distributions of two groups.

For manuscripts utilizing custom algorithms or software that are central to the research but not yet described in published literature, software must be made available to editors and reviewers. We strongly encourage code deposition in a community repository (e.g. GitHub). See the Nature Research guidelines for submitting code & software for further information.

Data

Policy information about availability of data

All manuscripts must include a data availability statement. This statement should provide the following information, where applicable:

- Accession codes, unique identifiers, or web links for publicly available datasets
- A list of figures that have associated raw data
- A description of any restrictions on data availability

The authors declare that all the data supporting the findings of this study are available within the paper and its supplementary information files. Primary antibodies and the nucleotide sequence for each shRNA used in this study are described in Methods. Source data are provided with this paper.

Field-specific reporting

Please select the one below that is the best fit for your research. If you are not sure, read the appropriate sections before making your selection.

[x] Life sciences [ ] Behavioural & social sciences [ ] Ecological, evolutionary & environmental sciences

For a reference copy of the document with all sections, see nature.com/documents/nr-reporting-summary-flat.pdf

Life sciences study design

All studies must disclose on these points even when the disclosure is negative.

Sample size	The chosen sample size are based on the numbers used for previous publications, which is most optimal to generate statistically significant results.
Data exclusions	No samples or animals were excluded from the analyses
Replication	All experimental findings were reproduced as stated in figure legends. All additional replication attempts were successful.
Randomization	All samples/animals were randomly allocated to experimental groups and processed.
Blinding	Experiments were not blinded. However, we followed standard laboratory procedures of randomization. Each experiment was designed with proper controls, and samples for comparison were collected and analyzed under the same conditions.

Reporting for specific materials, systems and methods

We require information from authors about some types of materials, experimental systems and methods used in many studies. Here, indicate whether each material, system or method listed is relevant to your study. If you are not sure if a list item applies to your research, read the appropriate section before selecting a response.

Materials & experimental systems

n/a	Involved in the study	
[ ]	[x]	Antibodies
[ ]	[x]	Eukaryotic cell lines
[x]	[ ]	Palaeontology and archaeology
[ ]	[x]	Animals and other organisms
[ ]	[x]	Human research participants
[x]	[ ]	Clinical data
[x]	[ ]	Dual use research of concern

Methods

n/a	Involved in the study	
[x]	[ ]	ChIP-seq
[x]	[ ]	Flow cytometry
[x]	[ ]	MRI-based neuroimaging

Antibodies

Antibodies used	Antibodies to phospho-Tyr416-Src (1:1000, Cat. 6943), Src (1:1000, Cat. 2123), lipin-1 (Cat. 14906), phospho-Tyrosine (1:1000, Cat. 9411), β-tubulin (1:1000, Cat. 2128), phospho-p70 S6 Kinase (1:1000, Cat. 9205), S70 S6 Kinase (1:1000, Cat. 9202) and Ki-67 (1:400 for IHC, Cat. 12202) were purchased from Cell Signaling Technology. Antibodies to lipin-1 (1:1000 for IB, H-120; 1:50 for IF, B-12), HA (1:1000, Y11, F7), normal Rabbit IgG (1:5000, sc-2027) and c-Src (1:1000, B12) were obtained from Santa Cruz Biotechnology. Antibodies to Flag (1:5000, Cat. F2555 and SAB4200071) and Actin (1:5000, Cat. A1978) were purchased from Sigma. Anti-Calnexin (1:1000 for IB, 1:100 for IF, Cat. 10427-2-AP) and anti-GST (1:5000, Cat. 66001-2-Ig) antibodies were purchased from Proteintech. Anti-LPIN3 (1:500, Cat. LS-C339442) was from LSBio. Anti-SEL[illegible] (1:1000, EPT-1, Cat. H00085465-A01) was from Novus Biologicals. Donkey anti-rabbit IgG secondary antibody Alexa Fluor 488 (Diluted 1:100 in PBS, Cat. A21206), and donkey anti-mouse IgG secondary antibody Alexa Fluor 594 (Diluted 1:100 in PBS, Cat. A21203) were purchased from Thermo Fisher Scientific. The HRP-conjugated goat anti-Mouse IgG (Cat. 115-035-003, 1:5000 for IB) and HRP-conjugated goat anti-Rabbit IgG (Cat. 111-035-003, 1:5000 for IB) antibodies were purchased from Jackson ImmunoResearch.<br>The rabbit polyclonal phospho-Ser106-lipin-1 (1:1000 for IB), phospho-Tyr398-lipin-1 (1:500 for IB), phospho-Tyr413-lipin-1 (1:500 for IB) and phospho-Tyr795-lipin-1 (1:500 for IB, 1:50 for IHC) antibodies were produced by immunizing 6-month-old male New Zealand White Rabbit in our lab.
Validation	The rabbit polyclonal phospho-Ser106-lipin-1 for IB were validated in Li TY et al., 2018. The rabbit polyclonal phospho-Tyr398-lipin-1, phospho-Tyr413-lipin-1 and phospho-Tyr795-lipin-1 antibody for IB were validated in this paper. The rabbit polyclonal phospho-Tyr795-lipin-1 for IHC were validated in this paper. All other commercial antibodies were validated by manufacturers.

Eukaryotic cell lines

Policy information about cell lines

Cell line source(s)	SK-BR3, MCF-7, HEK293T, MDA-MB-231 and MDA-MB-468 were obtained from ATCC. MMTV mouse mammary tumour cell lines from G. Ouyang (Xiamen University, Fujian, China).
Authentication	The cell lines have not been authenticated recently.
Mycoplasma contamination	The cell lines have been validated to be free of mycoplasma contamination
Commonly misidentified lines (See ICLAC register)	No commercial misidentified cells were used.

Animals and other organisms

Policy information about studies involving animals; ARRIVE guidelines recommended for reporting animal research

Laboratory animals	FVB/N-Tg (MMTV-PyVT) 634Mul/J (PyVT, Stock No. 002374) and BALB/cByJ-Lpin1fld/J (Lpin1+/-, Stock No. 001592) were obtained from Jackson Laboratories. The nude mice,NOD-SCID and the FVB/N mice were purchased from SLAC company (Shanghai). 2-month-old Lpin1+/+ and Lpin1-/- female mice were used for carmine alum staining. 8-week-old female nude mice or FVB/N female mice were used for xenograft studies. 6-week-old FVB/N or NOD-SCID or Lpin1+/+ and Lpin1-/- female mice were used for lung metastasis colonization. 6-week-old female Lpin1-/- mice were used in AAV injection.
Wild animals	The study did not involve wild animals.
Field-collected samples	The study did not involve field-collected samples.
Ethics oversight	All animal procedures were performed with an approved protocol from the Institutional Animal Care and Use Committee at Xiamen University.

Note that full information on the approval of the study protocol must also be provided in the manuscript.

Human research participants

Policy information about studies involving human research participants

Population characteristics	Human research participants only includes female, all of whom received standard adjuvant radiotherapy and chemotherapy after surgery.
Recruitment	N/A
Ethics oversight	This study was conducted in accordance with the ethics principles of the Declaration of Helsinki and approved by the Research and Ethics Committee of Sun Yat-Sen University Cancer Center (Guangzhou, China). All patients provided written informed consent.

Note that full information on the approval of the study protocol must also be provided in the manuscript.
